# pTripleTREP – A vector for tightly controlled expression and purification of virulence factors in *Staphylococcus aureus*

**DOI:** 10.1186/s12934-025-02736-7

**Published:** 2025-05-20

**Authors:** Hannes Wolfgramm, Christopher Saade, Marco Harms, Larissa M. Busch, Josephine Lange, Maximilian Schedlowski, Kristin Surmann, Manuela Gesell Salazar, Christian Hentschker, Leif Steil, Stephan Michalik, Uwe Völker, Alexander Reder

**Affiliations:** 1 Interfaculty Institute of Genetics and Functional Genomics, Department of Functional Genomics, University Medicine Greifswald, Greifswald, Germany; 2 Institute of Immunology, University Medicine Greifswald, Greifswald, Germany

**Keywords:** Homologous expression, Protein purification, Expression vector, *Staphylococcus aureus*, Twin-Strep-tag, Tetracycline-inducible promoter, Magnetic beads

## Abstract

**Background:**

Recombinant proteins facilitate and contribute to detailed studies of the virulence mechanisms and pathophysiology of the major human pathogen *Staphylococcus aureus*. Of particular interest are secreted virulence factors. However, due to their potential toxicity and specific post-translational processing, virulence factors are difficult targets for heterologous protein production. Purified proteins with native conformation and adequate purity can therefore often only be achieved by elaborate multi-step purification workflows. While homologous expression in *S. aureus* theoretically offers a promising alternative in this regard, its application remains limited due to the lack of systems that ensure both tightly controlled expression and subsequent efficient purification.

**Results:**

To bridge this gap, we present pTripleTREP as a versatile expression vector for *S. aureus*, which enables the homologous expression and purification of staphylococcal virulence factors. It features a strong SigA-dependent staphylococcal promoter overlapped by three tetracycline responsive elements (TRE), which ensures tight repression under control conditions and high expression levels upon induction of the target gene. This allowed very precise controlled production of the exemplary targets, serine protease-like protein A (SplA) and B (SplB). A simple single-step protein purification workflow using a Twin-Strep-tag and Strep-Tactin^®^XT coated magnetic beads yielded endotoxin-free Spl samples with purities above 99%. Thereby, the homologous production host facilitates native secretion and maturation without the need to engineer the target gene sequence. Proper signal peptide cleavage and the corresponding enzymatic activity of the generated protein products were confirmed for SplA and B.

**Conclusion:**

The expression vector pTripleTREP adds an important element to the staphylococcal molecular toolbox, facilitating the tightly controlled homologous expression and rapid native purification of secreted staphylococcal virulence factors. The optimised architecture and genetic features of the vector additionally provide a solid background for further applications such as plasmid-based complementation or interaction studies. Thus, pTripleTREP will support research on the role of staphylococcal virulence factors, paving the way for future therapeutic strategies to combat this pathogen.

**Supplementary Information:**

The online version contains supplementary material available at 10.1186/s12934-025-02736-7.

## Background

*Staphylococcus aureus* is a major human pathogen associated with significant healthcare costs [1–4]. The investigation of its specific virulence mechanisms and the development of targeted treatments are therefore continually relevant. Purified proteins are of particular importance in this process, because they enable the investigation of selected virulence factors and their direct involvement in infection and inflammation, independent of side effects. However, purified proteins must satisfy strict criteria in terms of purity, maturity, and activity to ensure reliable results [5].

Selection of the most appropriate system for the expression and purification of a specific target protein is crucial [6, 7]. Although an increasing number of expression systems suitable for a wide range of host organisms is available nowadays [e.g. 8–11], the majority of proteins is still produced by heterologous expression in *Escherichia coli* and *Bacillus subtilis* [12–14]. These heterologous expression systems are associated with challenges including low yield, proteolytic instability, restrained function, and co-purification of contaminants [6, 15–17].

Contamination with lipopolysaccharides (LPS) is particularly critical when working with *S. aureus* virulence factors. LPS are ubiquitous in Gram-negative bacteria [18]. Therefore, inadvertent co-purification is likely when protein production is performed in commonly used hosts such as *E. coli*, particularly due to the high affinity of LPS for poly-histidine tags [19], which are widely used for protein purification. As strong trigger for the human immune system, LPS can distort the results of immunological assays of *S. aureus* virulence factors. Its removal is a non-negligible challenge in protein purification [19–22]. However, each purification step reduces the protein yield and potentially the biological activity of the target protein [23]. One approach to mitigate LPS contamination is to secrete the target protein into extracellular space. Purification of target proteins from bacterial supernatant decrease the contaminant burden in the crude sample, reduces the proteolytic degradation of target proteins and streamlines the process by eliminating the need for cell disruption [24, 25]. However, the in silico prediction of optimal signal peptides for secretion of specific recombinant proteins is not trivial and the optimal signal peptide sequence varies strongly between different expression hosts, as well as between different target proteins produced in the same host [26, 27]. Accordingly, even well-established secretion systems are not necessarily the best choice for purifying a protein with many unknown parameters.

Homologous gene expression represents an alternative strategy. Nevertheless, due to the pathogenic nature of *S. aureus*, there are currently only a limited number of tools available for gene expression and subsequent target protein purification in this organism [28, 29]. None of these provide sufficiently controllable expression of the target gene. Some major vector systems were introduced based on pOS1 [30, 31] or pCN [32]. To our knowledge, the best adapted plasmid-based expression systems for *S. aureus* at the moment are pTSSCm [29], which allows the non-inducible expression of poly-histidine-tagged fusion proteins, and pRAB11BD [33], which allows the anhydrotetracycline (aTc) inducible expression of non-tagged proteins.

**Fig. 1:**
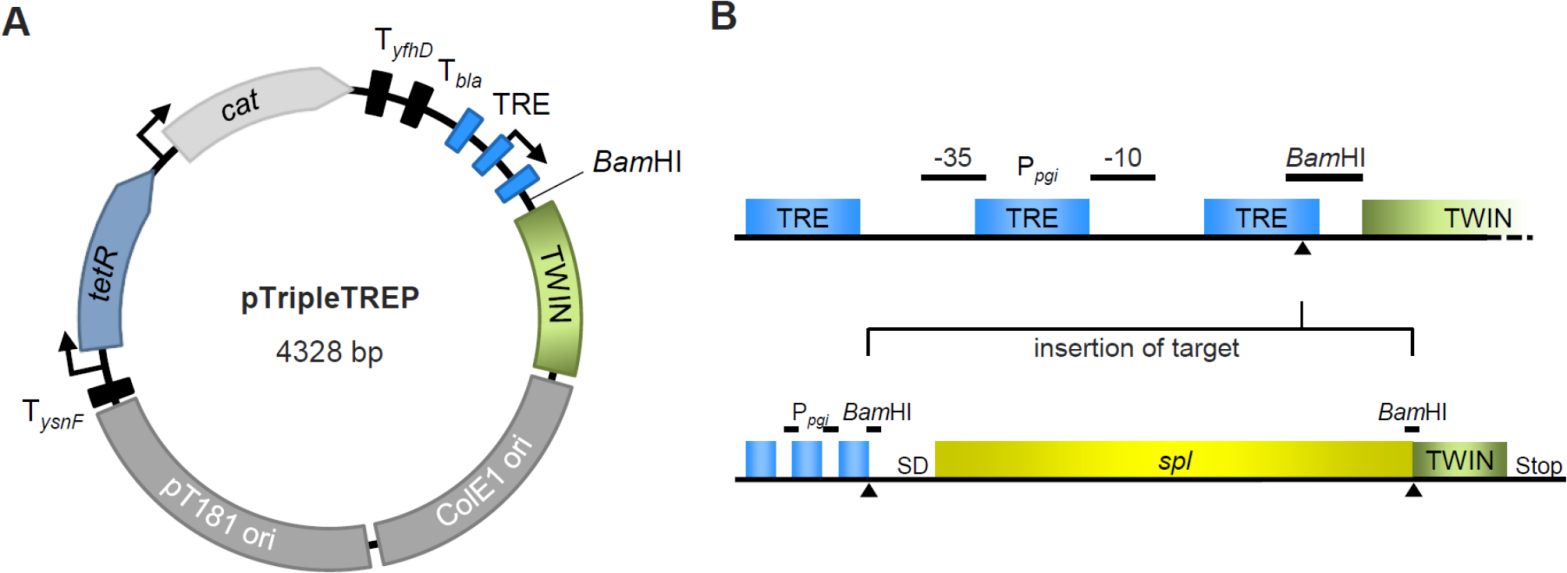
Schematic illustration of the expression vector pTripleTREP. (**A**) Representation of pTripleTREP with key features highlighted. Backbone components including origins of replication for *E. coli* (ColE1) and *S. aureus* (pT181) as well as chloramphenicol resistance cassette (*cat*) are shown in dark and light grey, respectively. Regulation is facilitated by an optimized TetR system (blue) consisting of the constitutively expressed repressor (*tetR*) and a susceptible promotor with tetracycline responsive elements (TRE). A Twin-Strep-tag (green) for single-step purification becomes translationally fused to targets cloned in the unique *Bam*HI site. Transcriptional terminators (T) are shown as black rectangles. (**B**) Detailed visualisation of the cloning site with the regulated target promoter (-10 and − 35 region indicated), unique *Bam*HI restriction site (black triangles) and Twin-Strep-tag. Target genes (yellow) can be inserted *via* restriction-ligation or SLIC (sequence and ligase independent cloning)

In our study, we present pTripleTREP, an innovative and versatile expression vector system for *S. aureus*. pTripleTREP was designed by integrating well-known, functionally proven elements from existing plasmid systems with newly defined functional components (Fig. 1A). The main components are (i) a newly described variant of the ColE1 replicon for plasmid replication in the cloning host *E. coli*, (ii) the pT181 replicon for plasmid replication in *S. aureus*, (iii) a chloramphenicol resistance cassette based on the chloramphenicol acetyltransferase gene (*cat*) as a universal selection marker, (iv) the *tetR* gene encoding the tetracycline repressor TetR combined with (v) an inducible strong SigA-type *S. aureus* target promoter containing three tetracycline responsive elements (TRE) to control gene expression of the cloned target gene (Fig. 1B) and, finally, (vi) a sequence encoding the Twin-Strep-tag for a straightforward single-step purification of the target protein. The resulting advantages of the pTripleTREP system for the purification of staphylococcal virulence factors - particularly in terms of procedure, purity and correct post-translational processing - are exemplified by expressing and purifying *S. aureus* serine protease-like proteins (Spls). Expression of *splA* and *splB* was tightly regulated, with a complete transcriptional repression under control conditions and a strong induction after addition of the inducer aTc. Upon induction, expression and purification of the target proteins, high protein yields and purity were obtained. Moreover, the modular design of pTripleTREP offers the opportunity to use the vector system in a variety of additional applications in *S. aureus*.

## Materials and methods

### Construction of pTripleTREP

The backbone of pTripleTREP was constructed in three major steps. All of these steps were performed by sequence and ligase independent cloning (SLIC) with the In-Fusion^®^ Snap Assembly Master Mix and competent *E. coli* Stellar™ cells (both Takara Bio, Japan) according to the manufacturer’s protocol. Transformed cells were grown overnight at 37 °C on LB agar plates containing 5 µg/mL chloramphenicol. Bacterial strains and plasmids used in this study are listed in Table 1.

**Table 1:** Bacterial strains and plasmids used in this study

Bacterial strains	Relevant genotype/ characteristics	Reference
*E. coli* Stellar™	HST08 derivate; *F–*,* endA1*,* supE44*,* thi-1*,* recA1*,* relA1*,* gyrA96*,* phoA*,* Φ80d lacZΔ M15*,* Δ(lacZYA-argF) U169*,* Δ(mrr-hsdRMS-mcrBC)*,* ΔmcrA*,* λ–*	Takara Bio, Japan
*S. aureus* RN4220	NCTC8325-4 derivate; defective in *hsdR*-, *sauUSI*-, *agrA*-, *essC*-, *mntH*-	[34]
*S. aureus* HG001	NCTC8325 derivate with restored in RsbU activity (RsbU^+^)	[35]
**Plasmids**		
pJL-sar-GFP	Reporter gene vector for *S. aureus;* ColE1 replicon, pT181 replicon, Amp^R^, Erm^R^, P_sarAP1_-*gfp*	[36]
pIMAY	Vector for allelic exchange in staphylococci; P_help_-*cat*	[37]
pTripleTREP	Expression vector; mutated ColE1 replicon, pT181 replicon, P_help_-*cat*, *tetR*, P_TRE_–*Bam*HI–Twin-Strep-tag	This study
pTripleTREP_splA.wt	*splA* wild-type sequence inserted in *Bam*HI site of pTripleTREP	This study
pTripleTREP_splA.mut	Point mutated *splA* sequence in pTripleTREP_splA.wt; codes for catalytically inactive SplA	This study
pTripleTREP_splB.wt	*splB* wild-type sequence inserted in *Bam*HI site of pTripleTREP	This study
pTripleTREP_splB.mut	Point mutated *splB* sequence in pTripleTREP_splB.wt; codes for catalytically inactive SplB	This study
pTripleTREP_clpX	*clpX* sequence inserted in pTripleTREP lacking the Twin-Strep-tag	This study

First, the two replicons were assembled with the chloramphenicol resistance cassette and the *tetR* gene. The ColE1 replicon was amplified from pJL-sar-GFP [36] including the upstream *bla* terminator with the primers mini_pUC(Cm)_ori_for and colE1_pTripleTREP_rev. The pT181 replicon was also amplified from pJL-sar-GFP in a first PCR with the primers pT181_pJL_for and pT181_pJL_rev, followed by a second PCR with pT181_pJL_for and pT181_pJL_pTripleTREP_rev. The chloramphenicol resistance cassette was amplified from pIMAY [37] with the primers Cm_pTripleTREP_for and Cm_for_mini_pUC(Cm)_rev. The *tetR* gene was manually codon optimized and synthesised including the staphylococcal *pgi* promoter sequence (GeneScript, Netherlands). The gene synthesis product was amplified with the primers TetR_pTripleTREP_in_for and TetR_pTripleTREP_in_rev. In this basic construct, the newly designed P_TRE_ promoter, followed by a *Bam*HI restriction site, and the Twin-Strep-tag sequence were inserted. Both elements were generated by megaprimers (P_TRE_: pTRE_pTripleTREP_in_for/rev, Twin-Strep-tag: TwStr_pTripleTrep_in_for/rev). In a PCR refill reaction, the complementary 3’ ends of the megaprimers (~ 30–40 bp) anneal to each other and serve simultaneously as primer and template for the completion of a double-stranded fragment. Plasmid linearization for the insertion of these fragments was performed with the primers pTripleTREP_linear_TwStr_for and pTripleTREP_linear_pTER_rev. In a last step, two additional terminators were inserted upstream of the *tetR* gene (*ysnF* terminator) and downstream of the chloramphenicol resistance cassette (*yfhD* terminator). While the *ysnF* terminator was assembled by the megaprimers ysnF_repC_in_for/rev (plasmid linearization with pTri_term_repC_for/rev), the *yfhD* terminator was amplified from *B. subtilis* 168 chromosomal DNA with the primers yfhD_Cm_in_for/rev (plasmid linearization with Cm_Term_in_for/rev_new). Information on the primer sequences are given in the supplements (Additional file 1: Table S1). The vector (Fig. 1A) is licensed by the ProTec Diagnostics GmbH (Germany).

### Clone selection and analysis of the ColE1 mutation

To select correct clones, solitary colonies were picked from the cloning plate, inoculated in 5 mL LB medium (Lennox; Sigma-Aldrich, MO USA) containing 5 µg/mL chloramphenicol and cultivated overnight at 37 °C with orbital shaking at 220 rpm. From a late stationary culture, 20 OD units (OD_540nm_) were harvested at 8,000 xg for 5 min. Plasmid isolation was performed using the High Pure Plasmid Isolation Kit (Roche Diagnostics, Switzerland) according to the manufacturer’s instructions and plasmid DNA concentration was determined using a NanoDrop spectrophotometer (Thermo Fisher Scientific, MA USA). The plasmid sequence was checked by Sanger sequencing (Eurofins Genomics Europe, Germany) and analysed using the Geneious Prime software version 2023.0.1 (Biomatters, New Zealand).

The influence of the identified mutation in the ColE1 replicon was analysed using empty plasmids that did not contain any target gene, only differing with regard to the ColE1 mutation. The relative amount of plasmid per OD unit was determined as described above. The predicted influence on the secondary structure of RNAI and RNAII was calculated using the mfold web server version 3.6 (mfold_util 4.7; [38]) with default settings. Structure plots and free energy values were taken from the output section.

### Generation of expression constructs

For the generation of expression constructs, the native genes *splA* and *splB* were amplified from *S. aureus* USA300 chromosomal DNA with primers splA_4_Trap_C-strep_for/rev and splB_4_Trap_C-strep_for/rev, respectively, and inserted in the *Bam*HI linearized vector pTripleTREP using the In-Fusion^®^ Snap Assembly Master Mix (Takara Bio, Japan). Additionally, a mutated version of each gene was generated coding for a catalytically inactive protease with an alanine substituting the serine of the catalytic triad [39]. This was derived by linearizing the plasmid with 5’ overlapping primers carrying respective nucleotide substitutions (splA_S189A_for/rev and splB_S193A_for/rev, respectively; Additional file 1: Table S2) followed by re-circularization. The resulting expression vectors pTripleTREP_splA.wt, pTripleTREP_splA.mut, pTripleTREP_splB.wt and pTripleTREP_splB.mut were verified by sequencing.

The expression vectors were transformed in *S. aureus* RN4220 [34] using the electroporation protocol described by Augustin and Götz [40]. Briefly, bacterial cells were washed repeatedly with 10% glycerol solution to become electrocompetent. Approximately 500 ng purified plasmid DNA were transformed by an electric pulse of 1 kV, 100 Ω and 25 µFd with an electrode gap of 1 mm. The cell suspension was taken up in SMMP medium, incubated at 37 °C for 6 h and finally plated on TSB agar plates containing 5 µg/mL chloramphenicol for overnight cultivation at 37 °C.

### Induction of gene expression and protein synthesis

All cultivation steps for protein production were carried out in liquid TSB medium containing 5 µg/mL chloramphenicol, incubated in a water bath at 37 °C with linear shaking for aerobic cultivation. An exponential pre-culture of *S. aureus* RN4220 with the respective pTripleTREP expression vector was used to inoculate a 200 mL main culture to a starting OD_540nm_ of 0.05. Target gene expression was induced in the early transient phase when cultures reached an optical density of OD_540nm_ 3.5 by the addition of anhydrotetracycline (aTc). In a comparison of different induction concentrations of aTc between 50 and 500 ng/mL, we confirmed that the commonly used final concentration of 200 ng/mL aTc is also optimal for pTripleTREP. The induction was followed by sampling immediately before and 30 min, 60 min and 120 min after the addition of aTc. Samples were immediately cooled down to 0 °C in liquid nitrogen, centrifuged for 10 min at 5,000 xg and 4 °C and cell free culture supernatants as well as bacterial pellets were stored separately at -80 °C. For protein purification, the complete culture was harvested 120 min after induction with aTc as described above using 50 mL tubes.

### RNA isolation and Northern blot analysis

Bacterial cell pellets corresponding to 16 OD units were resuspended in 200 µL of lysis solution (4 M guanidine-thiocyanate, 0.025 M Na-acetate pH 5.2, 10% N-lauroylsarcosinate) and dripped into a Teflon vessel filled with liquid nitrogen and an 8 mm diameter steel ball. Mechanical disruption of the cells was achieved by shaking the vessel for 3 min at 2,600 rpm in a mixer mill (Retsch, Germany). The disrupted cells were resuspended in 3 mL of lysis solution, aliquoted to 1 mL and frozen in liquid nitrogen. The RNA isolation was performed according to the acid phenol extraction described in Majumdar et al. [41]. The dried RNA pellets were dissolved in RNase-free water and all aliquots per sample were pooled again. An amount of 3 µg of total RNA per sample lane was used for Northern Blot analysis.

For quantitative detection of Northern Blot signals, fluorescence detection was used according to the protocol provided by ProTec Diagnostics (Germany; https://www.protec-diagnostics.com/shop/rna-true-marker4000-31/document/46). In principle, blotted RNA targets are detected by biotin labelled probes and a secondary fluorescent reagent coupled to streptavidin. In this study, probes against *cat* and *splB* were used, generated by primer pairs Cm_pIMAY_NOR_for/Cm_pIMAY_T7_rev and splB_for/splB_rev_T7, respectively (Additional file 1: Table S3) in combination with RNA-TRUE Dye700 (ProTec Diagnostics). Fluorescence detection was performed using an Odyssey^®^ CLx imaging system (LICORbio Biosciences, NE USA) in dual channel mode at 700 and 800 nm. RNA quality and equal loading per lane was checked by methylene blue staining of the blot after detection.

### SDS-PAGE and Western Blot analysis

The level of secreted target protein was evaluated by silver stained SDS-PAGE gels and Western Blots of precipitated culture supernatant. Therefore, sodium chloride was added to 300 µL of harvested supernatant (final concentration: 100 mM) and proteins were precipitated by the addition of 4 volumes of pure acetone overnight at -20 °C. Precipitated proteins were collected by centrifugation for 1 h at 17,000 x g and 4 °C and washed with 500 µL of 80% acetone (v/v). The air-dried protein pellets were resuspended in 100 µL 20 mM HEPES (pH 8.0) containing 1% SDS and stored at -80 °C. Denaturing SDS-PAGE was performed in 4 to 12% Bis-Tris NuPAGE™ gels (Thermo Fisher Scientific).

For silver staining, the separated proteins were fixed with fixation solution (50% methanol, 12% acetic acid) for 30 min. Fixation solution was washed off twice with 50% ethanol. Gels were incubated with 0.02% sodium thiosulfate pentahydrate solution for 1 min followed by incubation with staining solution (0.2% silver nitrate, 0.014% formaldehyde) for 20 min. Protein bands were visualized by reducing the bound silver ions in sodium carbonate solution (6% sodium carbonate, 0.005% sodium thiosulfate pentahydrate, 0.009% formaldehyde). Throughout all steps, the gels were gently shaken and washed with distilled water between different incubation steps, unless stated otherwise.

For Western Blot analysis, proteins were blotted on a 0.45 μm low fluorescence PVDF membrane using the Trans-Blot^®^ Turbo™ Transfer System (both Bio-Rad Laboratories, CA USA) according to the manufacturer’s protocol. Proteins were fixed on the membrane by incubating the membrane in 100% methanol for 5 min. Western Blot detection was performed according to the ‘Near-Infrared (NIR) Western Blot Detection Protocol’ of LICORbio Biosciences using SplB specific antibodies (primary mouse-anti-SplB) detected with a secondary antibody labelled with the NIR fluorescence dye IRDye^®^ 680RD (IRDye^®^ 680RD goat-anti-mouse; LICORbio Biosciences) and CW800-coupled Strep-Tactin^®^XT (LICORbio Biosciences). The Strep-Tactin^®^XT-CW800 acts as anti-Twin-Strep-tag detection reagent and detects the Twin-Strep-tagged proteins produced from pTripleTREP. Fluorescence detection was performed using an Odyssey^®^ CLx imaging system (LICORbio Biosciences) in dual channel mode at 700 nm (680RD) and 800 nm (CW800).

### Protein purification with magnetic beads

Proteins were purified directly from the culture supernatant samples using magnetic MagStrep^®^ Strep-Tactin^®^XT beads (IBA Lifesciences, Germany). To meet the binding requirements, the pH of the supernatant samples was adjusted to 7.0 and 8.0 using NaOH. Equilibrated beads from 1 mL bead suspension were mixed with 40 mL culture supernatant in a 50 mL tube. The batch was mixed continuously for 30 min at room temperature on a rotary mixer. After a 30 s centrifugation pulse, tubes were placed on a magnetic rack to remove the supernatant (collected as “flow-through”). The binding step was repeated with further 40 mL culture supernatant. Bead-bound proteins were washed three times by adding 3 mL DPBS (PAN-Biotech, Germany) each, vortex briefly, centrifuge briefly, and discard PBS at the magnetic rack (collected as wash step 1 to 3). Proteins were eluted from the beads by three sequential elution steps with 333 µL 100 mM biotin in LPS-free PBS each (1 mL in total). Elution took place on a magnetic rack after 15 min incubation at 37 °C with frequent shaking. To check for residual proteins on the bead surface after elution, beads were resuspended in 2% SDS and heated for 5 min at 95 °C. The bead free fraction was finally collected after separation on the magnetic rack (collected as “SDS elution”).

### Analysis of integrity and purity

Purification fractions were first tested for target protein content and contaminating proteins by silver staining after SDS-PAGE and Western Blot analysis as described above. The gel was loaded with 12 µL of flow-through and washing fractions, respectively, and 3 µL of the pooled elution fraction.

Purity of the purified proteins was analysed by mass-spectrometry (MS). Protein concentration of the purified proteins was determined as described in Reder et al. [42] using a Micro BCA™ Protein Assay (Thermo Fisher Scientific). For MS analysis, 300 ng purified protein of each batch were prepared using the SP3 protocol as described in previous publications [43, 44], digested with 12 ng Trypsin/Lys-C mix (Promega Corporation, WI USA). LC-MS/MS analysis was conducted using an Orbitrap Exploris™ mass spectrometer (Thermo Fisher Scientific) coupled to an UltiMate™ 3000 RSLCnano HPLC (Thermo Fisher Scientific) in a data dependent mode. For more information, please refer to Table S4 and S5 (Additional file 1). The mass spectrometry proteomics data have been deposited to the ProteomeXchange Consortium *via* the PRIDE [45] partner repository with the dataset identifier PXD060310.

The raw data were analysed individually for each protein variant using the SpectroMine™ software (v4.5.240625.52329; Biognosys). Deviating from the default settings, search was performed semi-specific (free N-terminus) with only methionine oxidation set as variable modification. The protein database searched against included in each case (i) the specific tagged Spl variant, (ii) 2,570 proteins of *S. aureus* RN4220 (protein fasta of NCBI RefSeq NZ_CP101124.1, downloaded April 2023), and (iii) the MaxQuant (v2.0.3.0; [46]) contaminant list filtered for keratins and trypsin (110 entries). Processed data were filtered for a peptide q-value below 0.01 and protein groups with at least two identified peptides. Purity values were calculated as percentages based on the label-free peptide quantities of peptides assigned to the target protein or any RN4220 protein in the database. To verify correct processing of the target proteases, absence of the signal peptide was examined by mass spectrometric sequence coverage. The identified peptides were mapped to the sequence of the respective tagged Spls and corresponding quantity values were summed up for each amino acid position.

LPS content was determined by using the Endosafe^®^ Portable Test System (PTS™) and associated Limulus Amebocyte Lysate (LAL) test cartridges (Charles River Laboratories, USA), following the manufacturer’s protocol. To improve detection limits, samples were measured undiluted or at a 1:2 dilution in LPS-free PBS, with a final volume of 100 µL. After the setup was completed, 25 µL of the sample was loaded into each reservoir of the LAL cartridge.

### Protease activity assay

Protease activity was assessed using synthetic 7-amino-4-methylcoumarin (AMC) substrates, following the method described by Dubin et al. for SplB [47]. The substrate Ac-YLY-AMC (BioCat, Germany) was used to measure SplA activity, while Ac-VEID-AMC (PeptaNova, Germany) was used for SplB. The reaction mixture consisted of 90 µL of 25 µM substrate solution and 10 µL of 2.5 µM protease solution in PBS. Reactions were performed in triplicate in 96-well flat-bottom black polystyrene chimney plates (Greiner Bio-One, Germany) at 37 °C with constant shaking. AMC fluorescence was monitored at an excitation wavelength of 360 nm and an emission wavelength of 460 nm, with measurements taken from the top.

### Complementation of *clpX*

The potential of pTripleTREP for plasmid-based complementation was proved by complementing *clpX* in *S. aureus* strain HG001 Δ*clpX*. The complementation plasmid pTripleTREP_clpX lacking the Twin-Strep-tag was constructed by linearizing pTripleTREP with the primers pTripleTREP_linear_TwStr_for and pTripleTrep_clpX_rev_long and inserting the native *clpX* sequence amplified from *S. aureus* HG001 chromosomal DNA with the primers clpX_TS_SD_for and clpX_Term_pTripleTrep_rev (primer sequences are given in additional file 1: Table S2) as described above. Transformation of pTripleTREP_clpX in HG001 Δ*clpX* was performed step-wise *via* electroporation with RN4220 as intermediate host. Strains HG001, HG001 Δ*clpX* and HG001 Δ*clpX* pTripleTREP_clpX were grown in TSB without antibiotic as described above. Deviating from the procedure for overexpression and protein purification, different concentrations of aTc (final concentration 0–50 ng/mL) were added to the medium directly from the start of the main culture. Cultures were grown for 2.5 h, then 16 OD units of each culture were harvested, immediately cooled down to 0 °C in liquid nitrogen and centrifuged for 10 min at 5,000 xg and 4 °C. Cell disruption was performed as described in Reder et al. [42].

To examine the ClpX protein levels in the different strains and conditions, a Western Blot analysis of 5 µg of each sample was performed with a polyclonal ClpX antiserum (primary rabbit anti-ClpX_*B. subtilis*_ [48]), and a secondary antibody labelled with the NIR fluorescence dye IRDye^®^ 800CW (IRDye^®^ 800CW goat-anti-rabbit). Fluorescence detection was performed using an Odyssey^®^ CLx imaging system (LICORbio Biosciences) in dual channel mode at 700 nm (680RD) and 800 nm (800CW).

### Data analysis

Data analysis and visualisation was conducted in R (v4.3.1; [49]) unless specified otherwise. The following R packages were used: tidyverse [50], readxl [51], janitor [52], ggbeeswarm [53], scales [54], ggrepel [55], patchwork [56].

## Results & discussion

### Design of the backbone

Since pTripleTREP (Fig. 1A) was explicitly designed for expression of virulence factors, the plasmid had to maintain stability when cloning potentially toxic targets – in both, the cloning host *E. coli* as well as the production host *S. aureus*. In this case, plasmid stability is primarily ensured by the strict repression of the target gene expression under non-inducing control conditions. Nevertheless, repression is affected by the plasmid copy number, hence the chosen replicon, as a high copy number can amplify even minimal leakage of repression [57]. At the same time, a high copy number is beneficial in both the cloning host, in order to easily obtain substantial amounts of plasmid DNA for genetic modification, and the production host, to achieve highest expression levels of the target protein. Accordingly, pTripleTREP was initially designed with the high-copy number pUC variant of the ColE1 replicon for *E. coli* from pUC19 [58] and the mid-copy number pT181 replicon for replication in *S. aureus* [32].

**Fig. 2:**
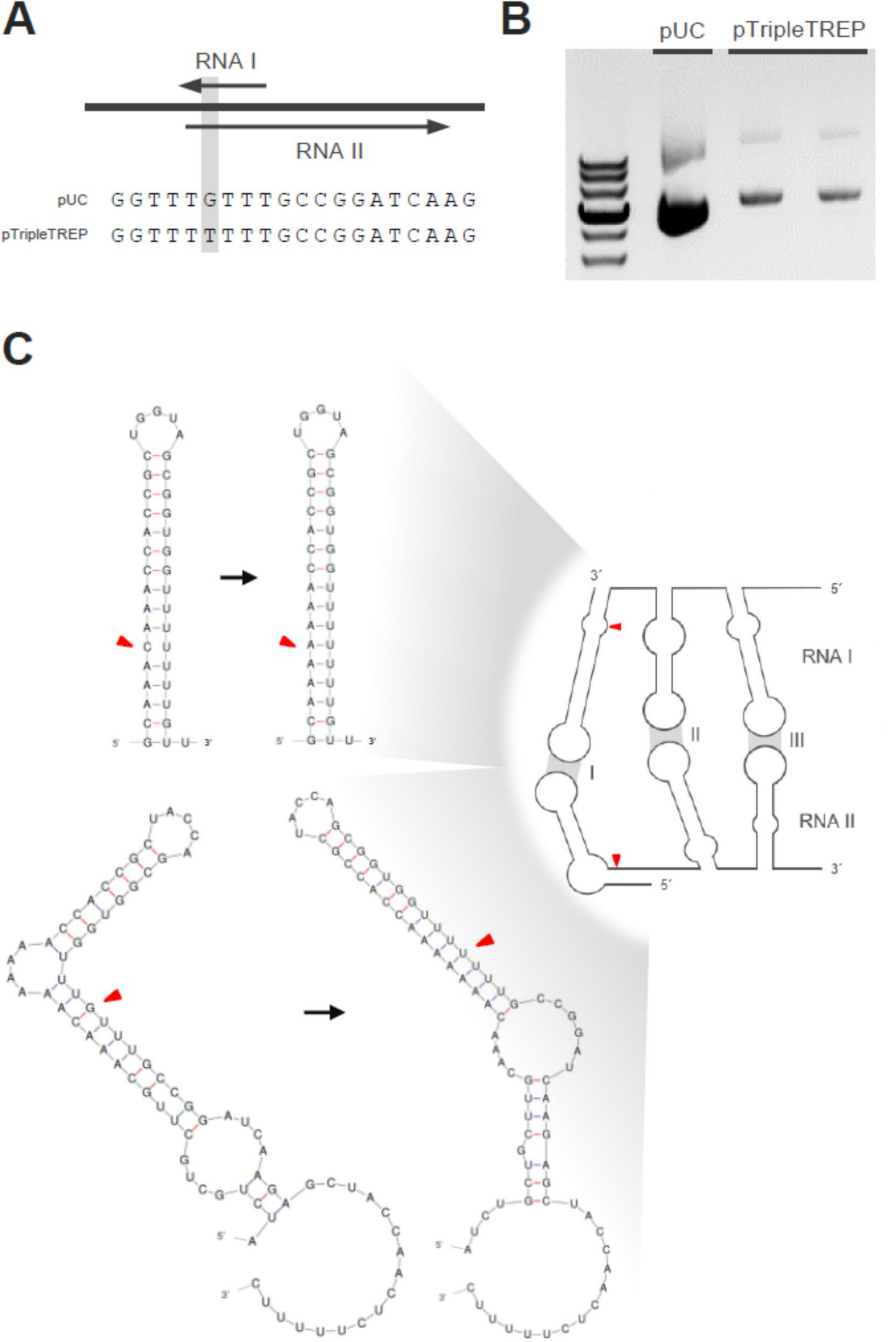
Mutation of the *E. coli* ColE1 origin of replication. **A**) Location of the G→T mutation in the ColE1 ori of pTripleTREP in comparison to pUC sequence. **B**) Plasmid yield of pTripleTREP with pUC ColE1 ori variant (labelled pUC) and mutated ColE1 ori variant (labelled pTripleTREP), respectively, isolated from 20 OD units of late stationary *E. coli* cells after selective growth. Plasmid yield is estimated by plasmid DNA amount determined *via* spectrophotometry (top) and by gel electrophoresis (bottom). **C**) Schematic representation of RNAI and RNAII interaction with the mutation site indicated by a red arrowhead. Detailed comparison of secondary structures of stem-loop I’ (top) and I (bottom), respectively. pUC ColE1 ori structure is shown on the left, pTripleTREP variant at the right. Location of the mutation site is indicated by a red arrowhead each. Secondary structures are predicted using the mfold web server version 3.6 (mfold_util 4.7; [38])

The pUC variant of the ColE1 replicon conveys a plasmid copy number of up to several hundred per cell [59]. This is due to a point mutation in the replication pre-primer RNAII compared to the original ColE1 replicon in pBR322 [60]. However, we observed impaired genetic stability and accumulation of mutations for certain target genes cloned in this high-copy ColE1 backbone already in *E. coli*. To overcome this issue, we screened multiple clones of the same transformation for mutations in the plasmid. From a single clone without mutations in the target gene, we finally isolated a ColE1 origin variant containing an additional point mutation (G→T) in the overlap of RNAI and RNAII coding sequence, more precisely in stem-loop I of RNAII and stem-loop I’ of the anti-sense RNAI (Fig. 2A). Interestingly, this variant occurs in a few other plasmids recorded on NCBI (Additional file 1: Table S5), but to our knowledge has not been previously described.

The observed mutation is associated with a 2.6-fold reduction in the amount of extracted plasmid from equal numbers of Stellar™ *E. coli* cells (420 ng to 160 ng plasmid DNA per OD unit; Fig. 2B), hence indicating a lower plasmid copy number. This reduction in plasmid copy number might be attributed to an enhanced interaction of RNAI and RNAII, as the mutation G→T eliminates a mismatch in the stem of stem-loops I and I’ (Fig. 2C). The predicted free energy decreases from − 21.10 kcal mol^− 1^ to -24.60 kcal mol^− 1^ for RNAI loop I and from − 28.20 kcal mol^− 1^ to -29.40 kcal mol^− 1^ for RNAII loop I’. This could support degradation of the pre-primer RNAII resulting in fewer replication initiation events (compare to Cesareni et al. [61]). Due to the observed improved genetic stability, we retained this mutated ColE1 variant in the pTripleTREP system.

The bacterial strategy of lowered plasmid copy number in response to the expression of toxins was described recently [62]. In general, the pUC variant of the ColE1 replicon apparently leads to strong heterogeneity in the plasmid copy number between cells [63] and was found to be segregational instable during heterologous protein expression [64]. Standley et al. improved segregational stability by introducing point mutations in RNAII (beyond the overlap to anti-sense RNAI) lowering the copy number of the ColE1 plasmids [64]. Given that the pTripleTREP variant of the ColE1 replicon similarly reduces plasmid copy number, it is likely that in addition to increased genetic stability, segregational stability is likewise enhanced.

The pT181 replicon conveys a medium copy number of approx. 25 plasmids per cell *via* a rolling-circle replication mechanism [32, 65]. While the copy-number is well-suited for target expression, rolling-circle replication introduces specific challenges that need to be considered: The stability of rolling-circle plasmids (i) is suspected to decrease significantly when the total plasmid size exceeds approx. 10 kb [66, 67] and (ii) can be impaired by replication-transcription collisions in open reading frames oriented against the direction of replication [68, 69]. Consequently, we substantially reduced the size of pTripleTREP (4.3 kb) compared to other existing systems by eliminating spacer sequences. This reduction in size facilitates stable integration of target DNA fragments up to 4.5 kb into pTripleTREP. As head-on conflicts between transcription and replication in highly expressed genes are reported to be more problematic for genetic integrity compared to co-directional conflicts [68, 70, 71], all genes in pTripleTREP were oriented in the direction of replication (Fig. 1A).

The incorporated resistance cassette is based on the chloramphenicol acetyltransferase gene (*cat*) as an universal selection marker that confers chloramphenicol resistance and allows selection for the plasmid in both, the cloning host *E. coli* as well as the production host *S. aureus* [72]. The cassette was derived from pIMAY [37], whereby a divergently oriented SigA promoter in the regulatory region of the P_help_ promoter [73] was eliminated.

### Design of the target regulation in pTripleTREP

To precisely adjust the timing and strength of target gene expression, we adopted and modified the widely used TetR system [74]. For Gram-positive bacteria, the TetR system is mainly based on the hybrid promoter P_xyl/tet_, first introduced in *B. subtilis* [75]. Derived from this architecture, there are three main steps in the development of plasmid-based TetR systems for use in *S. aureus*: (i) A P_xyl/tet_ version with one TRE was incorporated in pLZ113 [76] and pALC2073 [77], resulting in strong target gene expression under inducing conditions, but mediating a leaky repression in the absence of inducer. (ii) This basal target gene expression could be reduced in pRMC2 by adapting the *tetR* promoter to the *B. subtilis* consensus sequence [78]. (iii) The most recent widely used update was presented with pRAB11BD, which further reduced the basal target gene expression by facilitating a P_xyl/tet_ with two TRE and a chimeric TetR(BD) repressor [33]. In pTripleTREP, we have redesigned both of these components, the expression of *tetR* and the regulated target gene promoter.

**Fig. 3:**
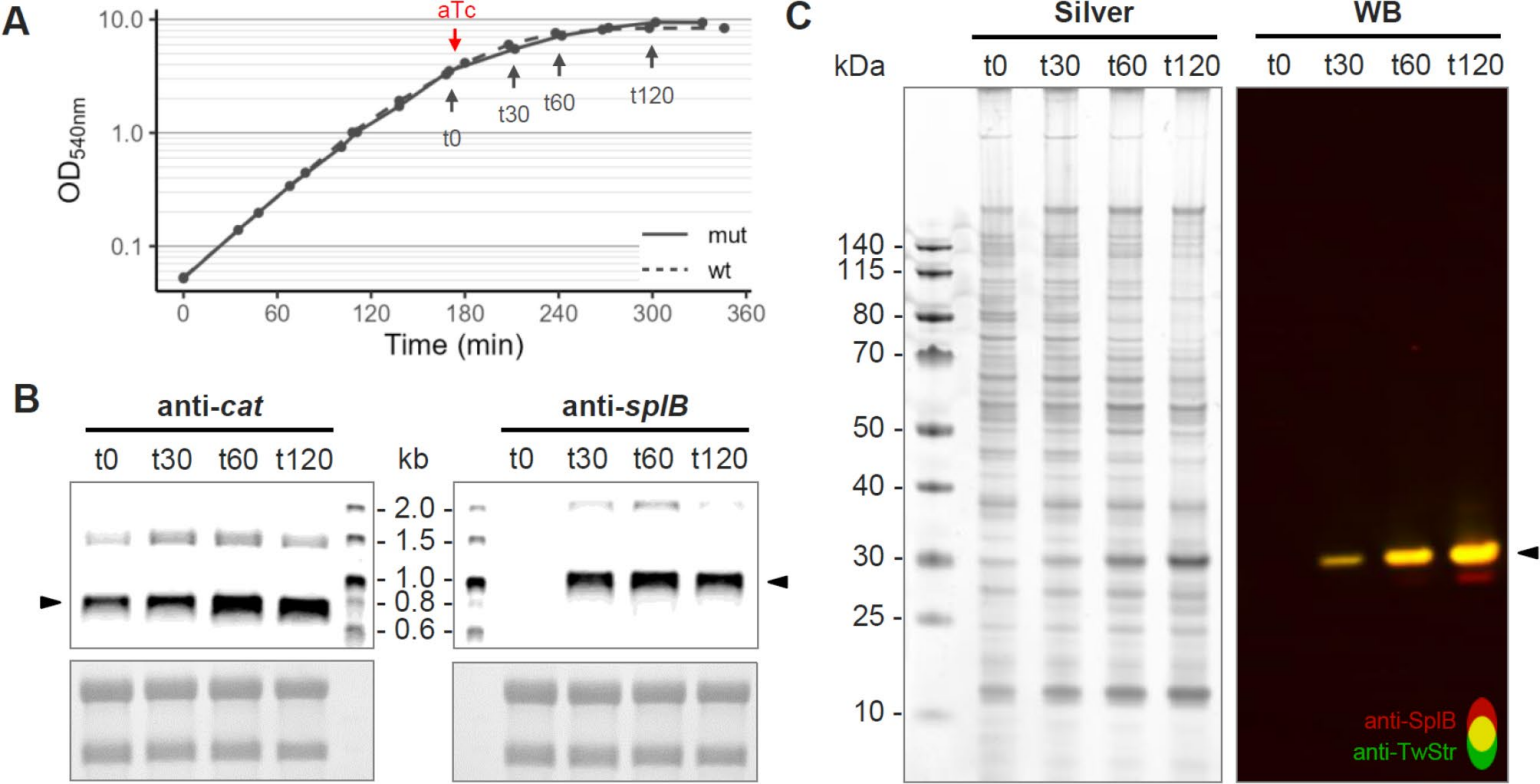
Analysis of overexpression of the exemplary target *slpB* after induction with 200 ng/mL anyhdrotetracyclin. (**A**) Growth curves of *S. aureus* RN4220 with pTripleTREP_splB.wt or pTripleTREP_splB.mut in 100 mL TSB (37 °C, 220 rpm). Time point of induction with 200 ng/mL anhydrotetracycline (aTc) and sampling time points are marked. (**B**) Northern Blot results depicting the expression level of the chloramphenicol selection marker (*cat*; left) and the target (*splB*, right) upon induction (marked with arrow heads). Probes are fluorescence labelled and detected at 800 nm, methylene blue staining of rRNA as loading control. (**C**) Silver stained SDS-PAGE and Western Blot (WB) of culture supernatant harvested at every time point show increase of secreted product after induction (molecular mass of Twin-Strep-tagged SplB.wt: ~29.1 kDa). An amount of 12 µL supernatant each was separated in a 4 to 12% gradient gel. The Western Blot signal was detected with primary mouse anti-SplB antibody (detection antibody anti-mouse-680RD; false coloured red) and Strep-Tactin^®^XT-CW800 detecting the Twin-Strep-tag (anti-TwStr; false coloured green), measured at 700 nm and 800 nm, respectively. Areas where both signals are superimposed appear in yellow

We optimized the *tetR* sequence to match codon usage and tRNA availability in *S. aureus* and placed it under the control of the strong, constitutively active SigA-dependent promoter P_*pgi*_ of the *S. aureus pgi* gene [79]. An appropriate level of *tetR* expression is crucial for balancing low basal target gene expression with minimal cellular stress, thus enabling a high dynamic range of regulation [65]. The P_*pgi*_ promoter fulfils these criteria and we did not observe any growth deficiency (Fig. 3A).

In addition, we designed a new TetR regulated promoter P_TRE_ harbouring the strong P_*pgi*_ core promoter elements in combination with three TREs, since the number of TRE in the promoter region is known to correlate with both the strength of the repression and the dynamic range of the system [33]. The TREs in pTripleTREP completely cover the promoter region with one TRE upstream of the − 35 region, one between the − 35 and − 10 regions, and one downstream of the − 10 region (Fig. 1B). As expected, no basal target gene expression from pTripleTREP under control conditions was observed (Fig. 3B). Since TetR repression has been shown to be stronger in Gram-negative bacteria than in Gram-positive bacteria [80], the strong repression demonstrated for pTripleTREP enables cloning of even toxic targets in the cloning host *E. coli* and their production in the expression host *S. aureus*. However, for problematic targets, a helper plasmid constitutively expressing *tetR* can further support repression of toxic target genes in *E. coli*.

In addition to the tight TetR-mediated repression, potential read-through from any transcriptional activity elsewhere in the plasmid backbone into the target gene sequence was eliminated by insertion of three transcriptional terminators. Of note, many of the terminators used in other systems are derived from *E. coli* and exhibit significant read-through in *S. aureus* [65]. Accordingly, we also observed a clear read-through in one of the first plasmid versions using only the pUC19 *bla* terminator (data not shown). To address this issue, we used the comprehensive *B. subtilis* transcriptome data of Nicolas et al. [81] to choose a set of terminators that fulfilled the criteria of (i) very high expression of the upstream gene, followed by (ii) a maximal transcriptional down-shift as well as (iii) a minimal read-through into the downstream region. Different terminators were tested for their potential termination activity by Northern Blotting (data not shown) and the two *B. subtilis* terminators of *ysnF* and *yfhD* were finally chosen to complete the terminator set in pTripleTREP (Fig. 1A). Many genetic elements can be easily transferred from *B. subtilis* to *S. aureus* [65]. In particular, transcriptional terminators are structurally similar between *B. subtilis* and *S. aureus* [82], although the free energy of *S. aureus* terminators is on average slightly lower than that of *B. subtilis* terminators [83].

The last TRE of P_TRE_ merges directly into a unique *Bam*HI restriction site, followed by the Twin-Strep-tag sequence [84]. This design allows the insertion of target genes without their stop codon *via* classical restriction and ligation cloning as well as sequence-and-ligase-independent cloning (SLIC; [85, 86]), both at the *Bam*HI site. Either way, the stop codon of the target gene must be replaced by the *Bam*HI site during target amplification to ensure translational fusion of the Twin-Strep-tag to the expressed protein. The 3` located *Bam*HI sequence is then translated into the two amino acid flexible linker between the target protein and the C-terminal Twin-Strep-tag. The target also has to be inserted with a ribosome binding site (RBS) – either the native or an engineered one. The choice of the RBS is left open in pTripleTREP, because it depends on many different factors including the specific target protein [87, 88]. The strength of homologous expression is, to exploit the evolved mechanisms that integrate translation, folding and secretion, which may favour the use of the native RBS for understudied targets.

### SplA and SplB as exemplary target proteins

The potential of pTripleTREP was demonstrated as proof-of-principle by expression of staphylococcal proteases *splA* and *splB*, subsequent protein purification, quality controls and functional validation. SplA and SplB are part of a cluster of secreted serine proteases in *S. aureus* [39, 89] with a suspected role in staphylococcal virulence [90]. In contrast to other secreted *S. aureus* proteases, the function of Spls is poorly understood, and although putative target cleavage sites have been defined [91], few physiological substrates are known to date [90, 92, 93].

As with many other secreted proteins, the activity of the Spls is dependent on correct post-secretion processing. The signal peptides of Spls contain an Ala-X-Ala motif indicating cleavage by the common type I signal peptidase (SPI) SpsB [39, 94]. Interestingly, unlike other secreted proteases, the mature form of Spls is released immediately after cleavage of the secretion signal - no extracellular, inactive pro-form is formed [95]. However, even single additional or missing amino acids at the N-terminus of the mature protease almost abolish the protease activity [95].

This specificity of the N-terminus is a challenge for heterologous expression systems. No remaining start methionine is permitted, nor is the correct signal peptide cleavage ensured in *E. coli* or *B. subtilis* ([27] and own unpublished results). Despite the strong conservation of the Ala-X-Ala motif in the SpsB substrate specificity, there is apparently a secondary substrate preference depending on the length and composition of the signal peptide [96–98]. Therefore, two genetic customisations were mainly used so far: (i) Transcriptional fusion with a signal peptide common in the production organism [47] or (ii) the introduction of a factor X cleavage site [99]. However, both methods require knowledge of correct processing of the target protein prior cloning and expression, as is the case for Spls. For new or less characterised targets, prediction of maturation is difficult [98]. pTripleTREP enables the homologous expression of targets and thus, the use of native secretion, processing and maturation in *S. aureus* without further analysis and genetic modification of the sequence.

### Controlled expression of Spls

Our optimized TetR system prevents even basal expression of target genes under control conditions. The native ORFs of the *S. aureus* HG001 *splA* and *splB* genes, respectively, each including the upstream RBS and the N-terminal signal peptide but excluding the STOP codon, were cloned into the *Bam*HI site of pTripleTREP (Fig. 1B). The expression pattern was exemplary analysed for *splB* wild-type. While the transcripts of the constitutively expressed genes *cat* (~ 0.75 kb) and *tetR* (as dicistronic *tetR-cat*-mRNA, ~ 1.5 kb) were present already before induction with aTc, the *splB* transcript (~ 1.0 kb and dicistronic *slpB*-*colE1* mRNA, ~ 1.9 kb) could only be detected after induction (Fig. 3B). The repression at the newly designed P_TRE_ target promoter and the transcriptional termination at the selected *B. subtilis* terminators thus successfully functioned as a continuous barrier against read-through leakage into the target gene. In addition to this strict repression under control conditions, the system enables stable expression after induction. Subsequent to the induction with a single dose of 200 ng/mL aTc, the *splB* transcription reached a stable and high level (Fig. 3B). On protein level, this leads to a strong increase of SplB (molecular mass of Twin-Strep-tagged SplB: ~29.1 kDa) in the culture supernatant, which accumulates to the most dominant protein therein (Fig. 3C). Same was true for the secretion of SplA (molecular mass of Twin-Strep-tagged SplA: ~28.6 kDa; Additional file 1: Fig. S1).

The dynamic range of an inducible expression system can be described by the induction factor (IF), which is defined as the fold change of a target-dependent signal from before induction to after induction. The IF achieved with the TetR system in pTripleTREP was at the very upper end of the commonly reported range. Following the commonly used design, the TetR system of pTripleTREP is based on the *tetR* of class (B) according to the study by Schnappinger et al. [100]. However, it has been shown that a chimeric repressor TetR(BD) consisting of a class (B) DNA-binding domain and a class (D) inducer binding domain can mediate a stronger induction of gene expression after induction [33]. Remarkably, the combination of the strong SigA-type promoter with three TREs in the target gene promoter P_TRE_ of pTripleTREP resulted in a comparably strong induction with the advantage of complete repression prior to induction. The *splB* transcript level before induction was in the background noise range of Northern Blot detection. Therefore, a meaningful IF could only be determined based on the Western Blot data. The comparison of SplB signal intensities before and 30 min after induction with 200 ng/ml aTc resulted in an IF of 320 (60 min: 840, 120 min: 1200), while the range of IFs considered as good stretches from about 25 [65] to 300 [33]. However, a generally valid IF that is comparable between different systems is not established so far, as it is strongly influenced by the choice of the reporter, the inducer concentration and the induction time, as well as the read-out system, especially its limits of detection [65, 74]. For example, the data from Helle et al. for a single system (pRAB11) yield an IF of about 2.6 in a fluorescence assay (pRAB11-gfp) but an IF of about 400 in a β-galactosidase assay (pRAB11-lacZ; [33]).

### Rapid purification of pure Recombinant Spls

In addition to strict expression control, pTripleTREP was designed to facilitate rapid, pure and native target protein purification based on the IBA Strep-Tactin^®^XT and Twin-Strep-tag system [84, 101]. This system offers a very high affinity on the one hand, which enables a fast and complete purification of the target proteins [84, 102] and on the other hand avoids the poly-histidine-tag with the associated increased co-purification of LPS [19]. Both properties enabled the high-quality purification of the exemplary targets SplA and SplB – in the native and a catalytically inactive form each – in a simple single-step procedure.

**Fig. 4:**
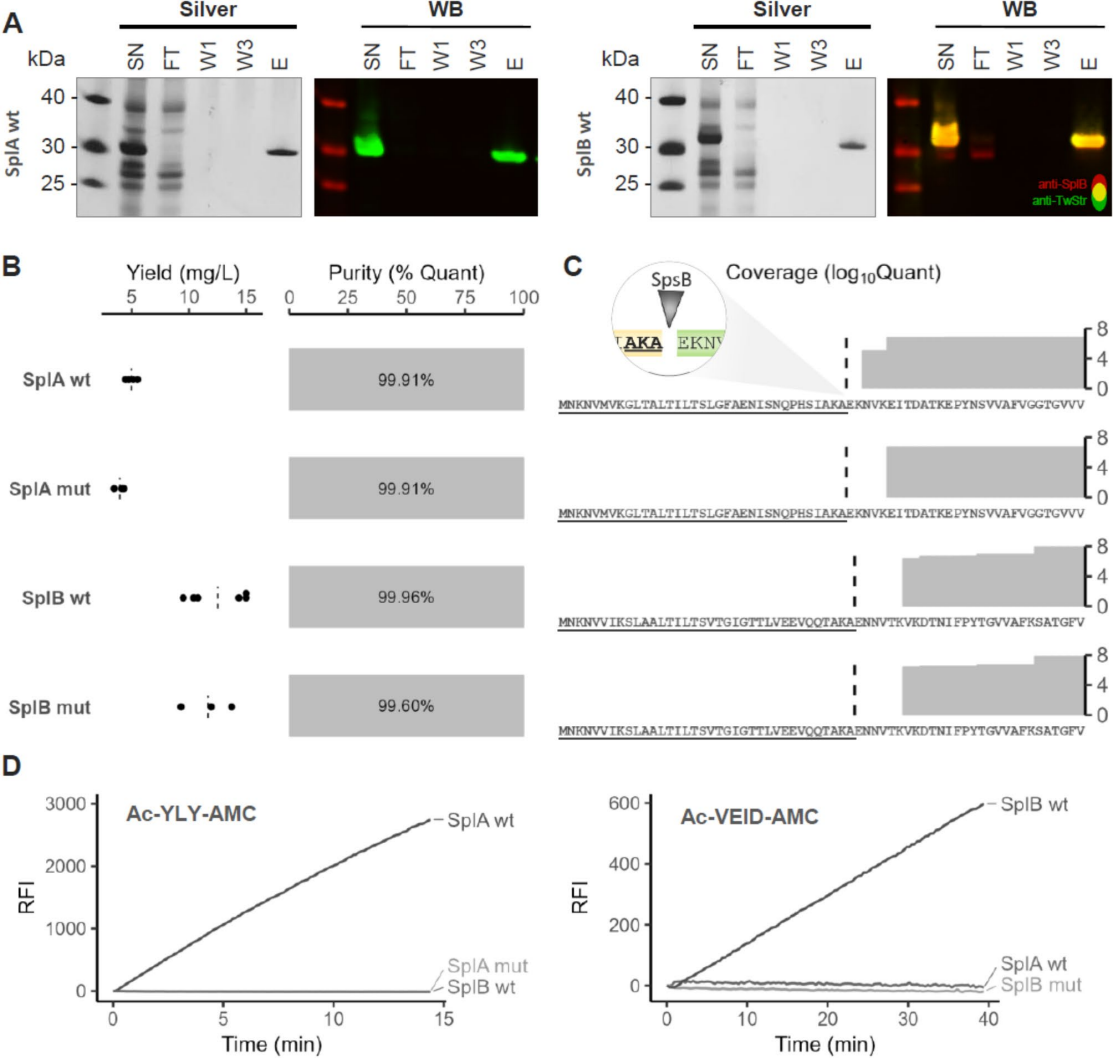
Quality characteristics of different Spl variants purified with the pTripleTREP system. All proteases (SplA and SplB, wild-type and enzymatically inactive mutant each) were expressed from the respective pTripleTREP version in *S. aureus* RN4220 cells and purified *via* C-terminal Twin-Strep-tag. Bacterial cultures were grown in TSB and induced with 200 ng/mL aTc when reaching OD_540nm_ 3.5. Supernatants were harvested 120 min after induction and targets were purified from 300 µL culture supernatant with 80 µL suspension of magnetic Strep-Tactin^®^XT beads. (**A**) Silver stained SDS-PAGE and Western Blot (WB) of culture supernatant (SN) and representative fractions – flow-through (FT), first wash (W1), third wash (W3) and elution (E) – of a SplA.wt (left) and a SplB.wt (right) purification. The Western Blot signal was detected with primary mouse anti-SplB antibody (detection antibody anti-mouse-680RD; false coloured red) and Strep-Tactin^®^XT-CW800 (anti-TwStr; false coloured green), measured at 700 nm and 800 nm, respectively. Areas where both signals are superimposed appear in yellow. (**B**) Average yield per 1 L starting culture and purity is shown for all samples. Purity was determined by LC-MS/MS and calculated as the percentage of target quantity to total quantity. (**C**) Correct N-terminal cleavage of the purified Spls by signal peptidase I SpsB was checked by peptide identification in LC-MS/MS. Summed quantities of identified peptides are mapped to the N-terminal protein sequence (signal peptide underlined). Coverage of full sequences in Fig. S3 (Additional file 1). (**D**) Activity assay of purified Spl samples with synthetic SplA-specific (Ac-YLY-AMC) and SplB-specific (Ac-VEID-AMC) substrates, respectively. Activity is measured as fluorescence increase of cleaved AMC proteolysis product detected at 460 nm

SDS-PAGE and Western Blot results demonstrate a tight and quantitative binding of all Twin-Strep-tagged Spl molecules to the beads and their release only in the elution step, no target protein appears in the wash fractions (Fig. 4A, Additional file 1: Fig. S2). Only a minor fraction of Spls remained bound to the beads after elution and were released upon heat denaturation in the presence of 2% SDS (Additional file 1: Fig. S2). Furthermore, we observed a strong separation of the target protein from other secreted proteins, since no proteins appear in the fractions beyond the flow-through. This also applies to the co-secreted SplB originating from the chromosomal *spl* operon, which is detected in the native supernatant and the flow-through by the SplB-specific antibody only but not the Strep-Tactin^®^XT (Fig. 4A right panel). This high selectivity after only one purification step is further underlined by the achieved purity. In the mass spectrometric analyses of the purified samples, the respective Spls always comprised more than 99% of the total protein quantities per sample (Fig. 4B). Additionally, the purified samples exhibited very low to no LPS contamination (< 0.2 EU/mL). Both quality controls fully meet the criteria for downstream in vitro and in vivo application including cell assays and animal experiments [20, 103, 104].

The purification was performed with different starting volumes of culture supernatant to determine the scalability. The extrapolated yield per 1 L supernatant ranged between 5.0 mg for SplA wild-type and 12.5 mg SplB wild-type (Fig. 4B). The amount of the inactive mutant was comparable each. The yield per 1 L starting culture thereby is largely comparable or even higher (SplB) than in other published Spl purifications, which also mirror the variance of yields depending on the Spl type [47, 91, 95, 99, 105]. Nevertheless, because these earlier purifications took place after heterologous expression in *E. coli* or *B. subtilis*, purity and correct maturation were achieved through several purification steps rather than one. The pTripleTREP system can therefore significantly reduce the time and materials required for a purification of *S. aureus* extracellular proteases.

Finally, the purified Spls were tested for the correct maturation and their proteolytic activity, which are closely linked [95]. In line with the mass spectrometric analysis, which shows the absence of the signal peptides for all purified Spl samples (Fig. 4C), a strong enzymatic activity of the wild-type proteases against their synthetic substrate was demonstrated in the activity assay (Fig. 4D). The catalytically inactive mutant forms indeed showed no activity against the synthetic substrates. Likewise, the wild-type SplA showed no activity against the SplB-specific substrate and vice versa. These results confirm the native maturation of the purified Spls and their associated substrate-specific catalytic activity [91, 105, 106]. The C-terminal Twin-Strep-tag did not interfere with the protein activity, as also shown in other publications [107, 108]. Due to the extremely high binding affinity of the Twin-Strep tag to Strep-Tactin^®^XT [101], pTripleTREP-derived proteins are ideally suited for protein interaction studies and the investigation of protein complexes using co-purification or pull-down assays [109–111]. The controllable expression allows a targeted application, so that questions about the role of staphylococcal virulence factors in pathophysiology can investigated under near-native conditions directly in the pathogenic organism *S. aureus*. If the tag needs to be removed completely, insertion of a Ni(II)-cleavage site could be considered [112]. For secreted *S. aureus* proteins with an unknown secretion signal, however, the homologous expression by pTripleTREP enables correct processing without genetic analysis or modification.

### Gradual adjustable complementation with pTripleTREP

Plasmid-based, inducible complementation is a useful tool, especially when a specific timing or level of the target gene expression is of interest or to avoid polar effects of chromosomal complementation. To showcase the potential use of pTripleTREP for such complementation studies in *S. aureus*, we complemented *clpX* in a HG001 Δ*clpX* deletion mutant with pTripleTREP_clpX. As already observed for the pTripleTREP_splB expression construct, no target protein was detected in the absence of aTc (molecular mass of ClpX: ~46.3 kDa; Additional file 1: Fig. S4), which highlights the strong repression of the target promoter in pTripleTREP under non-inducing conditions. The expression of the target gene is then induced in an aTc-dependent manner, starting from the lowest concentrations of 5 ng/ml aTc. At a final concentration between 20 and 50 ng/mL aTc in the medium, the ClpX amount already reaches roughly the level of the wild-type strain HG001 (Additional file 1: Fig. S4). The concentration necessary for physiological levels is in line with the aTc concentration used in recent complementation studies facilitated by pRAB11 [113, 114]. This brief insight into pTripleTREP as a tool for complementation studies indicates its potential beyond the homologous expression of secreted *S. aureus* proteins.

## Conclusion

With pTripleTREP, we present an expression vector system for homologous gene expression in *S. aureus* that combines the advantages of tightly controlled expression with high-purity, single-step purification of target proteins. This enables the rapid and simple production of staphylococcal proteins with native maturation and without the need for time-consuming endotoxin removal. The special attention paid to the tight repression of the system allows successful cloning of toxic gene products and makes the vector construct interesting not only for protein overexpression and purification, but also for physiological studies and complementation analyses. In the construction process, we were also able to uncover a mid-copy variant of the ColE1 origin of replication, which has already appeared in some other plasmid systems – apparently undetected due to evolutionary adaptation of the systems. Taken together, pTripleTREP adds an important building block to the genetic toolbox for *S. aureus* research.

## Electronic supplementary material

Below is the link to the electronic supplementary material.

Supplementary Material 1

## Data Availability

The mass spectrometry proteomics data have been deposited to the ProteomeXchange Consortium with the dataset identifier PXD060310. Further data generated or analysed during this study are included in this published article and its supplementary information files.

## References

[1] Nelson RE, Hatfield KM, Wolford H, Samore MH, Scott RDII, Reddy SC, et al. National estimates of healthcare costs associated with multidrug-resistant bacterial infections among hospitalized patients in the united States. Clin Infect Dis. 2021;72(Supplement1):S17–26. 33512523 10.1093/cid/ciaa1581 PMC11864165

[2] Adeiza SS, Islam MA, Shittu A. Global, regional, and National burdens: an overlapping meta-analysis on *Staphylococcus aureus* and its drug-resistant strains. One Health Bull. 2024;4(4):164.

[3] Rasigade JP, Dumitrescu O, Lina G. New epidemiology of *Staphylococcus aureus* infections. Clin Microbiol Infect. 2014;20(7):587–8. 24930666 10.1111/1469-0691.12718

[4] Lowy FD. *Staphylococcus aureus* infections. N Engl J Med. 1998;339(8):520–32. 9709046 10.1056/NEJM199808203390806

[5] Raynal B, Lenormand P, Baron B, Hoos S, England P. Quality assessment and optimization of purified protein samples: why and how? Microb Cell Factories. 2014;13(1):180. 10.1186/s12934-014-0180-6 PMC4299812 25547134

[6] Ahmad I, Nawaz N, Darwesh NM, ur Rahman S, Mustafa MZ, Khan SB, et al. Overcoming challenges for amplified expression of Recombinant proteins using *Escherichia coli*. Protein Expr Purif. 2018;144:12–8. 29180019 10.1016/j.pep.2017.11.005

[7] Terpe K. Overview of bacterial expression systems for heterologous protein production: from molecular and biochemical fundamentals to commercial systems. Appl Microbiol Biotechnol. 2006;72(2):211–22. 16791589 10.1007/s00253-006-0465-8

[8] Fujino Y, Goda S, Suematsu Y, Doi K. Development of a new gene expression vector for *Thermus thermophilus* using a silica-inducible promoter. Microb Cell Factories. 2020;19(1):126. 10.1186/s12934-020-01385-2 PMC7282064 32513169

[9] Löfblom J, Rosenstein R, Nguyen MT, Ståhl S, Götz F. *Staphylococcus carnosus*: from starter culture to protein engineering platform. Appl Microbiol Biotechnol. 2017;101(23):8293–307. 28971248 10.1007/s00253-017-8528-6 PMC5694512

[10] Ortega C, Prieto D, Abreu C, Oppezzo P, Correa A. Multi-compartment and multi-host vector suite for recombinant protein expression and purification. Front Microbiol. 2018 Jun 27;9:1384. 10.3389/fmicb.2018.01384 PMC6030378 29997597

[11] Romero Pastrana F, Neef J, van Dijl JM, Buist G. A *Lactococcus lactis* expression vector set with multiple affinity tags to facilitate isolation and direct labeling of heterologous secreted proteins. Appl Microbiol Biotechnol. 2017;101(22):8139–49. 28971274 10.1007/s00253-017-8524-x PMC5656699

[12] Deo S, Turton KL, Kainth T, Kumar A, Wieden HJ. Strategies for improving antimicrobial peptide production. Biotechnol Adv. 2022;59:107968. 35489657 10.1016/j.biotechadv.2022.107968

[13] Yang H, Qu J, Zou W, Shen W, Chen X. An overview and future prospects of Recombinant protein production in *Bacillus subtilis*. Appl Microbiol Biotechnol. 2021;105(18):6607–26. 34468804 10.1007/s00253-021-11533-2

[14] Zhang ZX, Nong FT, Wang YZ, Yan CX, Gu Y, Song P, et al. Strategies for efficient production of Recombinant proteins in *Escherichia coli*: alleviating the host burden and enhancing protein activity. Microb Cell Factories. 2022;21(1):191. 10.1186/s12934-022-01917-y PMC9479345 36109777

[15] Bolanos-Garcia VM, Davies OR. Structural analysis and classification of native proteins from *E. coli* commonly co-purified by immobilised metal affinity chromatography. Biochim Biophys Acta BBA - Gen Subj. 2006;1760(9):1304–13. 10.1016/j.bbagen.2006.03.027 16814929

[16] Correa A, Oppezzo P. Overcoming the solubility problem in *E. coli*: Available approaches for recombinant protein production. In: García-Fruitós E, editor. Insoluble Proteins: Methods and Protocols. Methods in Molecular Biology. Humana Press, New York, NY. 2015;1258:27–44. 10.1007/978-1-4939-2205-5_2 25447857

[17] Kaur J, Kumar A, Kaur J. Strategies for optimization of heterologous protein expression in *E. coli*: roadblocks and reinforcements. Int J Biol Macromol. 2018;106:803–22. 28830778 10.1016/j.ijbiomac.2017.08.080

[18] Beutler B, Rietschel ET. Innate immune sensing and its roots: the story of endotoxin. Nat Rev Immunol. 2003;3(2):169–76. 12563300 10.1038/nri1004

[19] Mack L, Brill B, Delis N, Groner B. Endotoxin depletion of recombinant protein preparations through their preferential binding to histidine tags. Anal Biochem. 2014 Dec 1;466:83–8. 10.1016/j.ab.2014.08.020 25172132

[20] Bonhomme D, Cavaillon JM, Werts C. The dangerous liaisons in innate immunity involving recombinant proteins and endotoxins: Examples from the literature and the Leptospira field. Journal of Biological Chemistry. 2024 Jan 1;300(1):105506. 10.1016/j.jbc.2023.105506 PMC10777017 38029965

[21] Khan MRI, Thangarasu M, Kang H, Hwang I. Plant produced endotoxin binding Recombinant proteins effectively remove endotoxins from protein samples. Sci Rep. 2022;12(1):16377. 36180579 10.1038/s41598-022-20776-6 PMC9525263

[22] Schneier M, Razdan S, Miller AM, Briceno ME, Barua S. Current technologies to endotoxin detection and removal for biopharmaceutical purification. Biotechnol Bioeng. 2020;117(8):2588–609. 32333387 10.1002/bit.27362

[23] Che Hussian CHA, Leong WY. Factors affecting therapeutic protein purity and yield during chromatographic purification. Prep Biochem Biotechnol. 2024;54(2):150–8. 37233514 10.1080/10826068.2023.2217507

[24] Anné J, Economou A, Bernaerts K. Protein secretion in Gram-positive bacteria: From multiple pathways to biotechnology. In: Bagnoli F, Rappuoli R, editors. Protein and Sugar Export and Assembly in Gram-positive Bacteria: Current Topics in Microbiology and Immunology. Springer International Publishing, Cham. 2016;404:267–308. 10.1007/82_2016_49 27885530

[25] Quax WJ. Merits of secretion of heterologous proteins from industrial microorganisms. Folia Microbiol (Praha). 1997;42(2):99–103. 9306652 10.1007/BF02898715

[26] Brockmeier U, Caspers M, Freudl R, Jockwer A, Noll T, Eggert T. Systematic screening of all signal peptides from *Bacillus subtilis*: A powerful strategy in optimizing heterologous protein secretion in Gram-positive bacteria. J Mol Biol. 2006;362(3):393–402. 16930615 10.1016/j.jmb.2006.07.034

[27] Freudl R. Signal peptides for Recombinant protein secretion in bacterial expression systems. Microb Cell Factories. 2018;17(1):52. 10.1186/s12934-018-0901-3 PMC5875014 29598818

[28] Bobrovskyy M, Willing SE, Schneewind O, Missiakas D. EssH peptidoglycan hydrolase enables *Staphylococcus aureus* type VII secretion across the bacterial cell wall envelope. J Bacteriol. 2018;200(20). 10.1128/jb.00268-18. 10.1128/JB.00268-18 PMC6153663 30082459

[29] Schwendener S, Perreten V. New shuttle vector-based expression system to generate polyhistidine-tagged fusion proteins in *Staphylococcus aureus* and *Escherichia coli*. Appl Environ Microbiol. 2015;81(9):3243–54. 25747000 10.1128/AEM.03803-14 PMC4393442

[30] Bubeck Wardenburg J, Williams WA, Missiakas D. Host defenses against *Staphylococcus aureus* infection require recognition of bacterial lipoproteins. Proc Natl Acad Sci. 2006;103(37):13831–6. 16954184 10.1073/pnas.0603072103 PMC1564215

[31] Schneewind O, Missiakas D. Genetic manipulation of Staphylococcus aureus. Curr Protoc Microbiol. 2014;32:Unit–C93. 24510849 10.1002/9780471729259.mc09c03s32 PMC6249557

[32] Charpentier E, Anton AI, Barry P, Alfonso B, Fang Y, Novick RP. Novel cassette-based shuttle vector system for Gram-positive bacteria. Appl Environ Microbiol. 2004;70(10):6076–85. 15466553 10.1128/AEM.70.10.6076-6085.2004 PMC522135

[33] Helle L, Kull M, Mayer S, Marincola G, Zelder ME, Goerke C, et al. Vectors for improved tet repressor-dependent gradual gene induction or Silencing in *Staphylococcus aureus*. Microbiology. 2011;157(12):3314–23. 21921101 10.1099/mic.0.052548-0

[34] Kreiswirth BN, Löfdahl S, Betley MJ, O’Reilly M, Schlievert PM, Bergdoll MS, et al. The toxic shock syndrome exotoxin structural gene is not detectably transmitted by a prophage. Nature. 1983;305(5936):709–12. 6226876 10.1038/305709a0

[35] Herbert S, Ziebandt AK, Ohlsen K, Schäfer T, Hecker M, Albrecht D, et al. Repair of global regulators in *Staphylococcus aureus* 8325 and comparative analysis with other clinical isolates. Infect Immun. 2010;78(6):2877–89. 20212089 10.1128/IAI.00088-10 PMC2876537

[36] Liese J, Rooijakkers SHM, van Strijp JAG, Novick RP, Dustin ML. Intravital two-photon microscopy of host-pathogen interactions in a mouse model of *Staphylococcus aureus* skin abscess formation. Cell Microbiol. 2013;15(6):891–909. 23217115 10.1111/cmi.12085

[37] Monk IR, Shah IM, Xu M, Tan MW, Foster TJ. Transforming the untransformable: Application of direct transformation to manipulate genetically *Staphylococcus aureus* and *Staphylococcus epidermidis*. Novick RP, editor. mBio. 2012;3(2):e00277–11. 10.1128/mBio.00277-11 PMC3312211 22434850

[38] Zuker M. Mfold web server for nucleic acid folding and hybridization prediction. Nucleic Acids Res. 2003;31(13):3406–15. 12824337 10.1093/nar/gkg595 PMC169194

[39] Reed SB, Wesson CA, Liou LE, Trumble WR, Schlievert PM, Bohach GA et al. EI Tuomanen editor 2001 Molecular characterization of a novel Staphylococcus aureus Serine protease Operon. Infect Immun 69 3 1521–7. 10.1128/IAI.69.3.1521-1527.2001 PMC98051 11179322

[40] Augustin J, Götz F. Transformation of *Staphylococcus epidermidis* and other Staphylococcal species with plasmid DNA by electroporation. FEMS Microbiol Lett. 1990;66(1–3):203–7. 10.1016/0378-1097(90)90283-v 2182373

[41] Majumdar D, Avissar YJ, Wyche JH. Simultaneous and rapid isolation of bacterial and eukaryotic DNA and RNA: a new approach for isolating DNA. Biotechniques. 1991;11(1):94–101. 1720004

[42] Reder A, Hentschker C, Steil L, Gesell Salazar M, Hammer E, Dhople VM, et al. MassSpecPreppy—An end-to-end solution for automated protein concentration determination and flexible sample digestion for proteomics applications. Proteomics. 2024;24(9):2300294. 10.1002/pmic.202300294 37772677

[43] Blankenburg, S; Hentschker, C; Nagel, A; Hildebrandt, P; Michalik, S; Dittmar, D; Surmann, K; Völker, U. Improving proteome coverage for small sample amounts: An advanced method for proteomics approaches with low bacterial cell numbers. Proteomics 2019;19(23):1900192. 10.1002/pmic.201900192 31532911

[44] Ganske A, Busch LM, Hentschker C, Reder A, Michalik S, Surmann K, et al. Exploring the targetome of IsrR, an iron-regulated sRNA controlling the synthesis of iron-containing proteins in Staphylococcus aureus. Front Microbiol. 2024 Jul 5;15:1439352. 10.3389/fmicb.2024.1439352 PMC11257911 39035440

[45] Perez-Riverol Y, Bandla C, Kundu DJ, Kamatchinathan S, Bai J, Hewapathirana S, et al. The PRIDE database at 20 years: 2025 update. Nucleic Acids Res. 2025 Jan 6;53(D1):D543–53. 10.1093/nar/gkae1011 PMC11701690 39494541

[46] Cox J, Mann M. MaxQuant enables high peptide identification rates, individualized p.p.b.-range mass accuracies and proteome-wide protein quantification. Nat Biotechnol. 2008;26(12):1367–72. 19029910 10.1038/nbt.1511

[47] Dubin G, Stec-Niemczyk J, Kisielewska M, Pustelny K, Popowicz GM, Bista M, et al. Enzymatic activity of the *Staphylococcus aureus* splb Serine protease is induced by substrates containing the sequence Trp-Glu-Leu-Gln. J Mol Biol. 2008;379(2):343–56. 18448121 10.1016/j.jmb.2008.03.059

[48] Gerth U, Kirstein J, Mostertz J, Waldminghaus T, Miethke M, Kock H, et al. Fine-Tuning in regulation of Clp protein content in *Bacillus subtilis*. J Bacteriol. 2004;186(1):179–91. 14679237 10.1128/JB.186.1.179-191.2004 PMC303445

[49] R Core Team. R: A language and environment for statistical computing [Internet]. Vienna, Austria: R foundation for statistical computing; 2024. Available from: https://www.R-project.org/

[50] Wickham H, Averick M, Bryan J, Chang W, McGowan LD, François R, et al. Welcome to the tidyverse. J Open Source Softw. 2019;4(43):1686.

[51] Wickham H, Bryan J, readxl. Read Excel Files [Internet]. 2023. Available from: https://github.com/tidyverse/readxl

[52] Firke S. janitor: Simple Tools for Examining and Cleaning Dirty Data [Internet]. 2023. Available from: https://github.com/sfirke/janitor

[53] Clarke E, Sherrill-Mix S, Dawson C. ggbeeswarm: Categorical Scatter (Violin Point) Plots [Internet]. 2023 [cited 2025 Jan 15]. Available from: https://github.com/eclarke/ggbeeswarm

[54] Wickham H, Seidel D. scales: Scale Functions for Visualization [Internet]. 2022 [cited 2025 Jan 15]. Available from: https://github.com/r-lib/scales

[55] Slowikowski K. ggrepel: Automatically Position Non-Overlapping Text Labels with ggplot2 [Internet]. 2023 [cited 2025 Jan 15]. Available from: https://github.com/slowkow/ggrepel

[56] Pedersen TL. patchwork: The Composer of Plots [Internet]. 2024. Available from: https://github.com/thomasp85/patchwork

[57] Anthony LC, Suzuki H, Filutowicz M. Tightly regulated vectors for the cloning and expression of toxic genes. J Microbiol Methods. 2004;58(2):243–50. 15234522 10.1016/j.mimet.2004.04.003

[58] Yanisch-Perron C, Vieira J, Messing J. Improved M13 phage cloning vectors and host strains: nucleotide sequences of the M13mp18 and pUC19 vectors. Gene. 1985;33(1):103–19. 2985470 10.1016/0378-1119(85)90120-9

[59] Lee CL, Ow DSW, Oh SKW. Quantitative real-time polymerase chain reaction for determination of plasmid copy number in bacteria. J Microbiol Methods. 2006;65(2):258–67. 16181694 10.1016/j.mimet.2005.07.019

[60] Lin-Chao S, Chen WT, Wong TT. High copy number of the pUC plasmid results from a Rom/Rop-suppressible point mutation in RNA II. Mol Microbiol. 1992;6(22):3385–93. 1283002 10.1111/j.1365-2958.1992.tb02206.x

[61] Cesareni G, Helmer-Citterich M, Castagnoli L. Control of ColE1 plasmid replication by antisense RNA. Trends Genet. 1991;7(7):230–5. 1887504 10.1016/0168-9525(91)90370-6

[62] Ruan S, Bourne CR. *Escherichia coli* cells evade inducible ParE toxin expression by reducing plasmid copy number. Microbiol Spectr. 2024;12(6):e03973–23. 38700352 10.1128/spectrum.03973-23 PMC11237751

[63] Jahn M, Vorpahl C, Hübschmann T, Harms H, Müller S. Copy number variability of expression plasmids determined by cell sorting and droplet digital PCR. Microb Cell Factories. 2016;15(1):211. 10.1186/s12934-016-0610-8 PMC5168713 27993152

[64] Standley MS, Million-Weaver S, Alexander DL, Hu S, Camps M. Genetic control of ColE1 plasmid stability that is independent of plasmid copy number regulation. Curr Genet. 2019;65(1):179–92. 29909438 10.1007/s00294-018-0858-0 PMC6309527

[65] Rondthaler SN, Sarker B, Howitz N, Shah I, Andrews LB. Toolbox of characterized genetic parts for *Staphylococcus aureus*. ACS Synth Biol. 2024;13(1):103–18. 38064657 10.1021/acssynbio.3c00325 PMC10805105

[66] Bron S, Luxen E, Swart P. Instability of Recombinant pUB110 plasmids in *Bacillus subtilis*: Plasmid-encoded stability function and effects of DNA inserts. Plasmid. 1988;19(3):231–41. 2852818 10.1016/0147-619x(88)90041-8

[67] Pluta R, Espinosa M. Antisense and yet sensitive: copy number control of rolling circle-replicating plasmids by small RNAs. Wiley Interdiscip Rev RNA. 2018;9(6):e1500. 30074293 10.1002/wrna.1500

[68] Mirkin EV, Mirkin SM. Mechanisms of transcription-replication collisions in bacteria. Mol Cell Biol. 2005;25(3):888–95. 15657418 10.1128/MCB.25.3.888-895.2005 PMC544003

[69] Wein T, Hülter NF, Mizrahi I, Dagan T. Emergence of plasmid stability under non-selective conditions maintains antibiotic resistance. Nat Commun. 2019;10(1):2595. 31197163 10.1038/s41467-019-10600-7 PMC6565834

[70] Vilette D, Ehrlich SD, Michel B. Transcription-induced deletions in plasmid vectors: M13 DNA replication as a source of instability. Mol Gen Genet MGG. 1996;252(4):398–403. 8879240 10.1007/BF02173004

[71] Srivatsan A, Tehranchi A, MacAlpine DM, Wang JD. Co-Orientation of replication and transcription preserves genome integrity. PLOS Genet. 2010;6(1):e1000810. 20090829 10.1371/journal.pgen.1000810 PMC2797598

[72] Roberts MC, Schwarz S. Tetracycline and chloramphenicol resistance mechanisms. In: Mayers D, Sobel J, Ouellette M, Kaye K, Marchaim D, editors. Antimicrobial Drug Resistance: Mechanisms of Drug Resistance. Springer International Publishing, Cham. 2017;1:231–43.

[73] Riedel CU, Monk IR, Casey PG, Morrissey D, O’Sullivan GC, Tangney M, et al. Improved luciferase tagging system for *Listeria monocytogenes* allows real-time monitoring *in vivo* and *in vitro*. Appl Environ Microbiol. 2007;73(9):3091–4. 17351089 10.1128/AEM.02940-06 PMC1892880

[74] Bertram R, Neumann B, Schuster CF. Status quo of *tet* regulation in bacteria. Microb Biotechnol. 2022;15(4):1101–19. 34713957 10.1111/1751-7915.13926 PMC8966031

[75] Geissendörfer M, Hillen W. Regulated expression of heterologous genes in *Bacillus subtilis* using the Tn*10* encoded *tet* regulatory elements. Appl Microbiol Biotechnol. 1990;33(6):657–63. 1369298 10.1007/BF00604933

[76] Zhang L, Fan F, Palmer LM, Lonetto MA, Petit C, Voelker LL, et al. Regulated gene expression in *Staphylococcus aureus* for identifying conditional lethal phenotypes and antibiotic mode of action. Gene. 2000;255(2):297–305. 11024290 10.1016/s0378-1119(00)00325-5

[77] Bateman BT, Donegan NP, Jarry TM, Palma M, Cheung AL. Evaluation of a Tetracycline-Inducible Promoter in *Staphylococcus aureus* In Vitro and In Vivo and Its Application in Demonstrating the Role of *sigB* in Microcolony Formation. Infect Immun. 2001;69(12):7851–7. 10.1128/IAI.69.12.7851-7857.2001 PMC98881 11705967

[78] Corrigan RM, Foster TJ. An improved tetracycline-inducible expression vector for *Staphylococcus aureus*. Plasmid. 2009;61(2):126–9. 18996145 10.1016/j.plasmid.2008.10.001

[79] Mäder U, Nicolas P, Depke M, Pané-Farré J, Debarbouille M, van der Kooi-Pol MM et al. *Staphylococcus aureus* transcriptome architecture: From laboratory to infection-mimicking conditions. Kearns DB, editor. PLOS Genet. 2016;12(4):e1005962. 10.1371/journal.pgen.1005962 PMC4818034 27035918

[80] Bertram R, Hillen W. The application of tet repressor in prokaryotic gene regulation and expression. Microb Biotechnol. 2008;1(1):2–16. 21261817 10.1111/j.1751-7915.2007.00001.x PMC3864427

[81] Nicolas P, Mäder U, Dervyn E, Rochat T, Leduc A, Pigeonneau N, et al. Condition-dependent transcriptome reveals high-level regulatory architecture in *Bacillus subtilis*. Science. 2012;335(6072):1103–6. 22383849 10.1126/science.1206848

[82] Unniraman S, Prakash R, Nagaraja V. Conserved economics of transcription termination in eubacteria. Nucleic Acids Res. 2002;30(3):675–84. 11809879 10.1093/nar/30.3.675 PMC100295

[83] de Hoon MJL, Makita Y, Nakai K, Miyano S. Prediction of transcriptional terminators in *Bacillus subtilis* and related species. PLOS Comput Biol. 2005;1(3):e25. 16110342 10.1371/journal.pcbi.0010025 PMC1187862

[84] Schmidt TGM, Batz L, Bonet L, Carl U, Holzapfel G, Kiem K, et al. Development of the Twin-Strep-tag^®^ and its application for purification of Recombinant proteins from cell culture supernatants. Protein Expr Purif. 2013;92(1):54–61. 24012791 10.1016/j.pep.2013.08.021

[85] Li C, Evans RM. Ligation independent cloning irrespective of restriction site compatibility. Nucleic Acids Res. 1997;25(20):4165–6. 9321675 10.1093/nar/25.20.4165 PMC147003

[86] Li MZ, Elledge SJ. Harnessing homologous recombination *in vitro* to generate Recombinant DNA via SLIC. Nat Methods. 2007;4(3):251–6. 17293868 10.1038/nmeth1010

[87] Li ZJ, Zhang ZX, Xu Y, Shi TQ, Ye C, Sun XM, et al. CRISPR-based construction of a BL21 (DE3)-derived variant strain library to rapidly improve Recombinant protein production. ACS Synth Biol. 2022;11(1):343–52. 34919397 10.1021/acssynbio.1c00463

[88] Salis HM, Mirsky EA, Voigt CA. Automated design of synthetic ribosome binding sites to control protein expression. Nat Biotechnol. 2009;27(10):946–50. 19801975 10.1038/nbt.1568 PMC2782888

[89] Kläui AJ, Boss R, Graber HU. Characterization and comparative analysis of the Staphylococcus aureus genomic island vSaβ: an in silico approach. J Bacteriol. 2019 Nov 15;201(22):e00777–18. 10.1128/JB.00777-18 PMC6805111 31451542

[90] Dasari P, Nordengrün M, Vilhena C, Steil L, Abdurrahman G, Surmann K et al. MJ Federle editor 2022 The protease splb of Staphylococcus aureus targets host complement components and inhibits complement-mediated bacterial opsonophagocytosis. J Bacteriol 204 1 e00184–21. 34633872 10.1128/JB.00184-21 PMC8765433

[91] Stach N, Karim A, Golik P, Kitel R, Pustelny K, Gruba N, et al. Structural determinants of substrate specificity of SplF protease from *Staphylococcus aureus*. Int J Mol Sci. 2021;22(4):2220. 33672341 10.3390/ijms22042220 PMC7926377

[92] Paharik AE, Salgado-Pabon W, Meyerholz DK, White MJ, Schlievert PM, Horswill AR. The Spl Serine proteases modulate *Staphylococcus aureus* protein production and virulence in a rabbit model of pneumonia. mSphere. 2016;1(5):e00208–16. 27747296 10.1128/mSphere.00208-16 PMC5061998

[93] Scherr F, Darisipudi MN, Börner FR, Austermeier S, Hoffmann F, Eberhardt M, et al. Alpha-1-antitrypsin as novel substrate for *S. aureus*’ Spl proteases – implications for virulence. Front Immunol. 2024 Nov 19;15:1481181. 10.3389/fimmu.2024.1481181 PMC11611844 39628483

[94] von Heijne G. Patterns of amino acids near signal-sequence cleavage sites. Eur J Biochem. 1983;133(1):17–21. 6852022 10.1111/j.1432-1033.1983.tb07424.x

[95] Pustelny K, Zdzalik M, Stach N, Stec-Niemczyk J, Cichon P, Czarna A, et al. Staphylococcal splb Serine protease utilizes a novel molecular mechanism of activation. J Biol Chem. 2014;289(22):15544–53. 24713703 10.1074/jbc.M113.507616 PMC4140910

[96] Dalbey RE, Wang P, van Dijl JM. Membrane proteases in the bacterial protein secretion and quality control pathway. Microbiol Mol Biol Rev. 2012;76(2):311–30. 22688815 10.1128/MMBR.05019-11 PMC3372248

[97] van Roosmalen M, Jongbloed JH, Dubois JYF, Venema G, Bron S, van Dijl JM. Distinction between major and minor *Bacillus* signal peptidases based on phylogenetic and structural criteria. J Biol Chem. 2001;276(27):25230–5. 11309398 10.1074/jbc.M102099200

[98] Schallenberger MA, Niessen S, Shao C, Fowler BJ, Romesberg FE. Type I signal peptidase and protein secretion in *Staphylococcus aureus*. J Bacteriol. 2012;194(10):2677–86. 22447899 10.1128/JB.00064-12 PMC3347181

[99] Popowicz GM, Dubin G, Stec-Niemczyk J, Czarny A, Dubin A, Potempa J, et al. Functional and structural characterization of Spl proteases from *Staphylococcus aureus*. J Mol Biol. 2006;358(1):270–9. 16516230 10.1016/j.jmb.2006.01.098

[100] Schnappinger D, Schubert P, Pfleiderer K, Hillen W. Determinants of protein–protein recognition by four helix bundles: changing the dimerization specificity of tet repressor. EMBO J. 1998;17(2):535–43. 9430644 10.1093/emboj/17.2.535 PMC1170403

[101] Yeliseev A, Zoubak L, Schmidt TGM. Application of Strep-Tactin XT for affinity purification of Twin-Strep-tagged CB2, a G protein-coupled cannabinoid receptor. Protein Expr Purif. 2017;131:109–18. 27867058 10.1016/j.pep.2016.11.006 PMC5406253

[102] Dammeyer T, Timmis KN, Tinnefeld P. Broad host range vectors for expression of proteins with (Twin-) *Strep*- Tag, His-tag and engineered, export optimized yellow fluorescent protein. Microb Cell Factories. 2013;12(1):49. 10.1186/1475-2859-12-49 PMC3680311 23687945

[103] de Marco A, Berrow N, Lebendiker M, Garcia-Alai M, Knauer SH, Lopez-Mendez B, et al. Quality control of protein reagents for the improvement of research data reproducibility. Nat Commun. 2021;12(1):2795. 33990604 10.1038/s41467-021-23167-z PMC8121922

[104] Remans K, Lebendiker M, Abreu C, Maffei M, Sellathurai S, May MM, et al. Protein purification strategies must consider downstream applications and individual biological characteristics. Microb Cell Factories. 2022;21(1):52. 10.1186/s12934-022-01778-5 PMC8991485 35392897

[105] Stec-Niemczyk J, Pustelny K, Kisielewska M, Bista M, Boulware KT, Stennicke HR, et al. Structural and functional characterization of SplA, an exclusively specific protease of *Staphylococcus aureus*. Biochem J. 2009;419(3):555–64. 19175361 10.1042/BJ20081351

[106] Pustelny K, Stach N, Wladyka B, Dubin A, Dubin G. Evaluation of P1’ substrate specificity of staphylococcal SplB protease. Acta Biochimica Polonica. 2014 Mar 20;61(1):149–52. 24649483

[107] Ringel P, Probst C, Dammeyer T, Buchmeier S, Jänsch L, Wissing J, et al. Enzymatic characterization of Recombinant nitrate reductase expressed and purified from *Neurospora crassa*. Fungal Genet Biol. 2015;80:10–8. 25914160 10.1016/j.fgb.2015.04.016

[108] Wang L, Fan M, Zeng C, Li W, Hu Q, Liu W, et al. Expression and purification of a rapidly degraded protein, TMEM8B-a, in mammalian cell line. Protein Expr Purif. 2018;151:38–45. 29886078 10.1016/j.pep.2018.06.002

[109] Liu X, Abad L, Chatterjee L, Cristea IM, Varjosalo M. Mapping protein–protein interactions by mass spectrometry. Mass Spectrometry Reviews. 2024 May 14;mas.21887. 10.1002/mas.21887 PMC11561166 38742660

[110] Müller VS, Jungblut PR, Meyer TF, Hunke S. Membrane-SPINE: an improved method to identify protein–protein interaction partners of membrane proteins *in vivo*. Proteomics. 2011;11(10):2124–8. 21472855 10.1002/pmic.201000558

[111] Yin M, Yan Z, Li X. Structural insight into the assembly of the type II secretion system pilotin–secretin complex from enterotoxigenic Escherichia coli. Nat Microbiol. 2018;3(5):581–7. 29632366 10.1038/s41564-018-0148-0

[112] Abd Elhameed HAH, Hajdu B, Balogh RK, Hermann E, Hunyadi-Gulyás É, Gyurcsik B. Purification of proteins with native terminal sequences using a Ni(II)-cleavable C-terminal hexahistidine affinity Tag. Protein Expr Purif. 2019;159:53–9. 30905870 10.1016/j.pep.2019.03.009

[113] Sierra R, Prados J, Panasenko OO, Andrey DO, Fleuchot B, Redder P, et al. Insights into the global effect on Staphylococcus aureus growth arrest by induction of the endoribonuclease MazF toxin. Nucleic Acids Res. 2020;48(15):8545–61. 32735661 10.1093/nar/gkaa617 PMC7470975

[114] Wu Y, Meng Y, Qian L, Ding B, Han H, Chen H, et al. The Vancomycin Resistance-Associated regulatory system VraSR modulates biofilm formation of *Staphylococcus epidermidis* in an *ica*-Dependent manner. mSphere. 2021;6(5). 10.1128/msphere.00641-21. 10.1128/mSphere.00641-21 PMC8550092 34550006

