# Supplementary material for: pTripleTREP – A vector for tightly controlled expression and purification of virulence factors in Staphylococcus aureus
Source: Microb Cell Fact. 2025 May 20;24:115. doi: 10.1186/s12934-025-02736-7 (PMC12090601; doi:10.1186/s12934-025-02736-7)
Supplement: Supplementary file 1 — Supplementary Material 1 [file 12934_2025_2736_MOESM1_ESM.docx]

**Supplementary information**

**pTripleTREP – A vector for tightly controlled expression and purification of virulence factors in *Staphylococcus aureus***

Hannes Wolfgramm^1^, Christopher Saade^2^, Marco Harms^1^, Larissa M. Busch^1^, Josephine Lange^1^, Maximilian Schedlowski^1^, Kristin Surmann^1^, Manuela Gesell Salazar^1^, Christian Hentschker^1^, Leif Steil^1^, Stephan Michalik^1^, Uwe Völker^1*^, Alexander Reder^1*^

^1^ Interfaculty Institute of Genetics and Functional Genomics, Department of Functional Genomics, University Medicine Greifswald, Greifswald, Germany

^2^ Institute of Immunology, University Medicine Greifswald, Greifswald, Germany

* corresponding author; [redera@uni-greifswald.de](mailto:redera@uni-greifswald.de); [voelker@uni-greifswald.de](mailto:voelker@uni-greifswald.de)


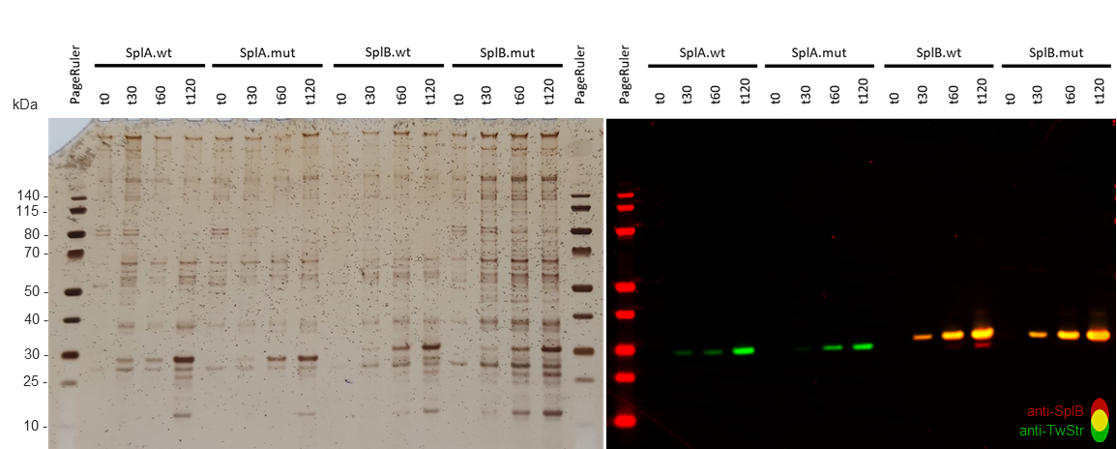


**Figure S1. SDS-PAGE and Western Blot of all purified Spl variants.** Silver stained SDS-PAGE (left) and Western Blot (right) of culture supernatant harvested at every time point show increase of secreted product after induction. An amount of 12 µL supernatant each was separated in a 4 to 12% gradient gel. The Western Blot signals were detected with primary mouse anti-SplB antibody (detection antibody anti-mouse-680RD; false coloured red) and Strep-Tactin®XT-CW800 (anti-TwStr; false coloured green), measured at 700 nm and 800 nm, respectively. Areas where both signals are superimposed appear in yellow. Twin-Strep-tagged SplA is only detected with Strep-Tactin®XT-CW800 (green; molecular mass: ~28.6 kDa), Twin-Strep-tagged SplB is detected with Strep-Tactin®XT-CW800 and mouse anti-SplB antibody (green + red = yellow; molecular mass: ~29.1 kDa).


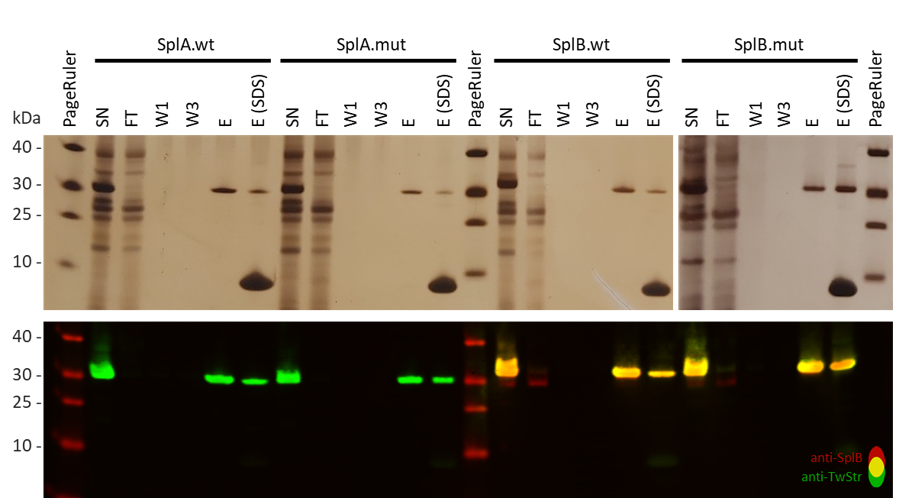


**Figure S2. SDS-PAGE and Western Blot of purification fractions from all purified Spl variants.** Silver stained SDS-PAGE (top) and Western Blot (bottom) of culture supernatant (SN) and representative fractions – flow-through (FT), first wash (W1), third wash (W3), elution (E) and SDS elution (E (SDS)) – of Spl purifications with magnetic Strep-Tactin®XT beads. 80 µL bead suspension were used with 300 µL culture supernatant of RN4220 pTripleTREP 120 min after induction with 200 ng/mL anhydrotetracycline (t120). Washing and elution were performed in 200 mM HEPES pH 8.0. The Western Blot signals were detected with primary mouse anti-SplB antibody (detection antibody anti-mouse-680RD; false coloured red) and Strep-Tactin®XT-CW800 (anti-Twin-Strep-tag; false coloured green), measured at 700 nm and 800 nm, respectively. Areas where both signals are superimposed appear in yellow. Twin-Strep-tagged SplA is only detected with Strep-Tactin®XT-CW800 (green; molecular mass: ~28.6 kDa), Twin-Strep-tagged SplB is detected with Strep-Tactin®XT-CW800 and mouse anti-SplB antibody (green + red = yellow; molecular mass: ~29.1 kDa).


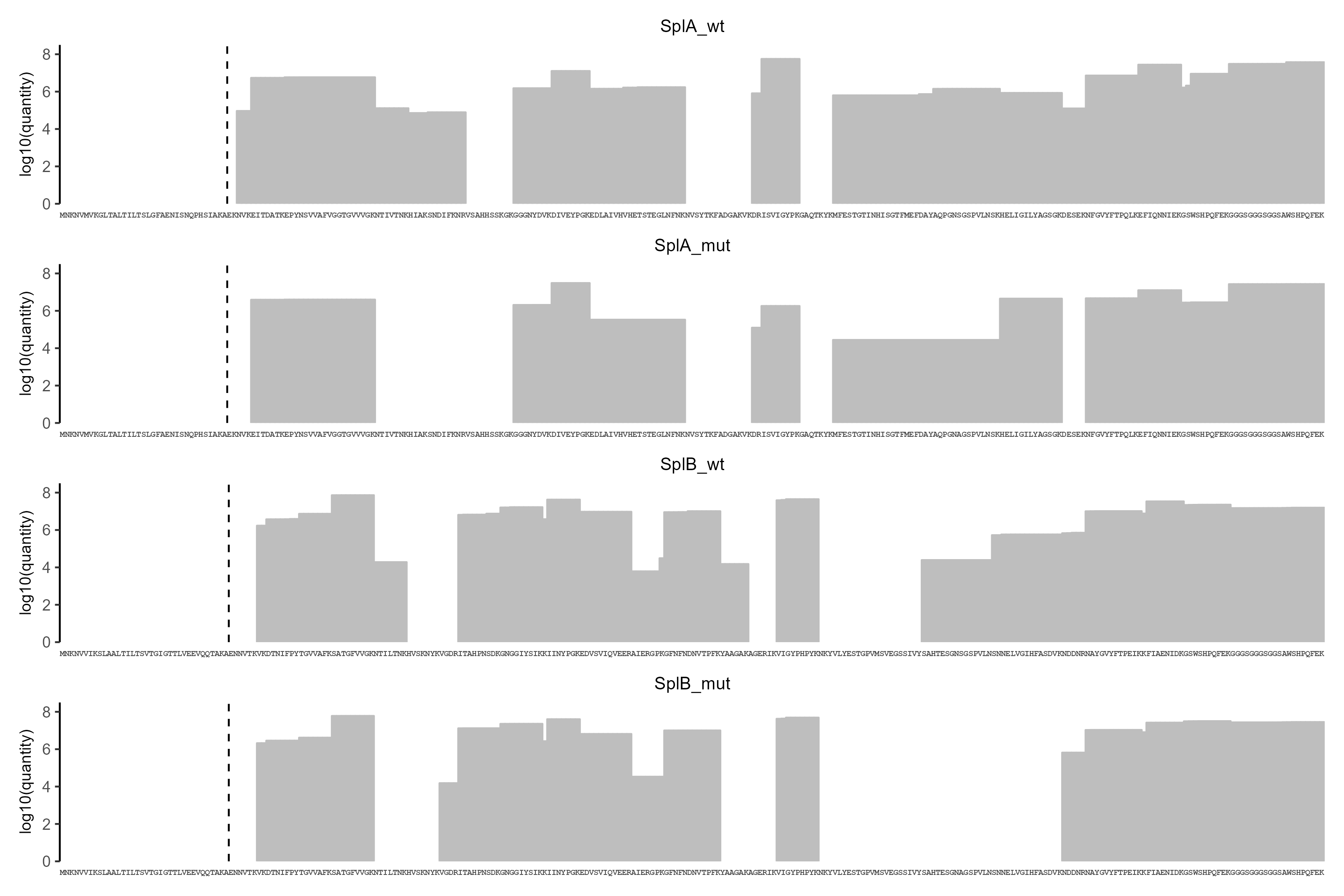


**Figure S3. Mass spectrometric sequence coverage of the purified Spls.** Purified Spl samples were analysed by LC-MS/MS in DDA mode and separately searched against the respective target sequence. Label-free quantities of identified peptides (q-value < 0.01) were summed per amino acid. The dashed line marks the expected signal peptide cleavage site (AXA↓).


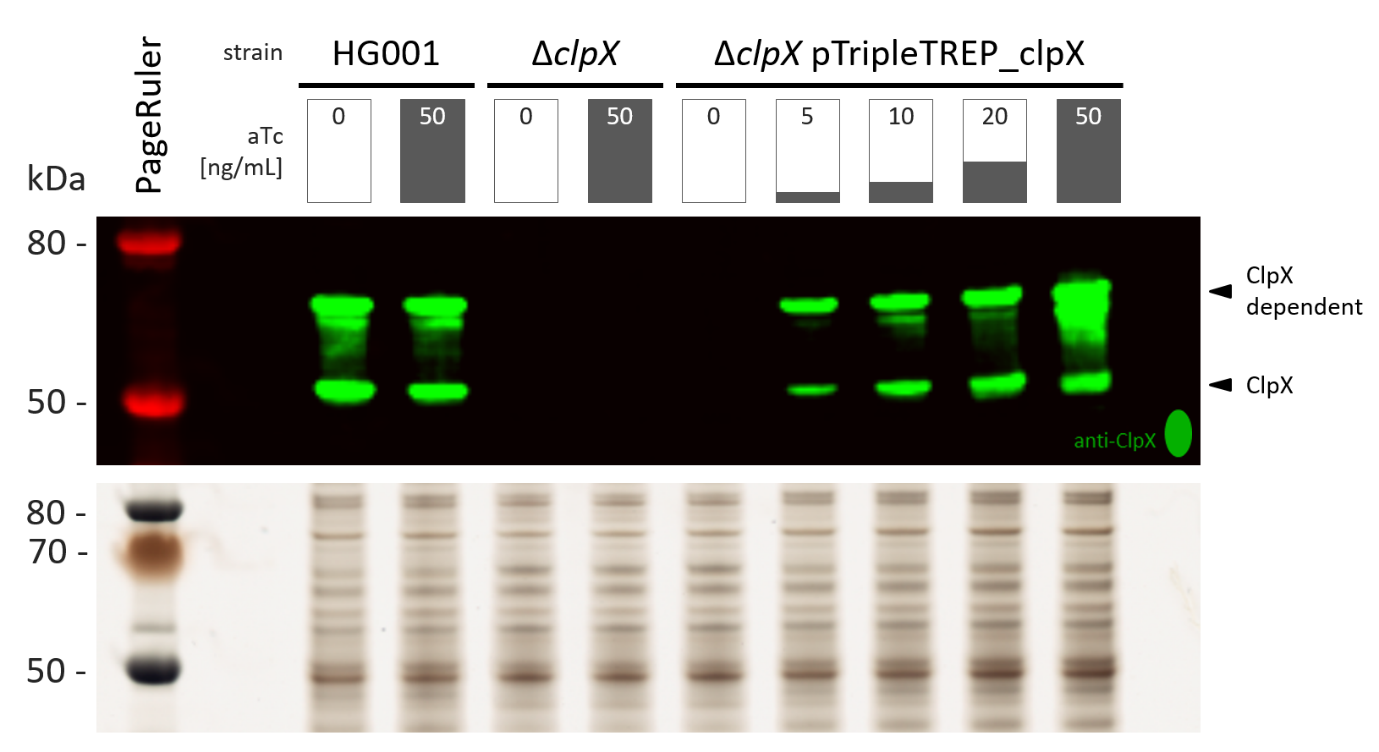


**Figure S4.** **Western Blot analysis of HG001 *clpX* complementation.** Western Blot (top) and silver stained SDS-PAGE (bottom) of 5 µg protein extracts from strains HG001, HG001 Δ*clpX*, and HG001 Δ*clpX* pTripleTREP_clpX grown for 2.5 hours in TSB containing different concentrations of aTc. In the complemented strain HG001 Δ*clpX* pTripleTREP_clpX, no ClpX is expressed when aTc is absent and levels increases with increasing aTc concentrations (molecular mass ClpX: ~46 kDa). The Western Blot signals were detected with primary polyclonal anti-ClpX antiserum (detection antibody anti-rabbit-800CW; false coloured green), measured at 800 nm. Signal at larger size (~70 kDa) indicates ClpX with covalently bound factors or posttranslational modifications (under investigation).

**Table S1. Primers for the construction of the expression vector pTripleTREP.** Primers were synthesized by Biolegio (Netherlands).

| Primer | Sequence (5’-3’) |
| --- | --- |
| pTripleTREP backbone |  |
| mini_pUC(Cm)_ori_for | ATAATATGAGATAATCTGTCAGACCAAGTTTACTC |
| colE1_pTripleTREP_rev | ATCGACGTCGGTAATACGGTTATCCACAGAATC |
| pT181_pJL_for | ATTACCGACGTCGATAGGAACAGCAACAAGAACAAC |
| pT181_pJL_rev | AACTCACGAAATTAGAATAGTTTAAATATATC |
| pT181_pJL_pTripleTREP_rev | AAACAACTCACGAAATTAGAATAG |
| Cm_pTripleTREP_for | AGGAAGCTAAATCCCATTATGCTTTGGCAG |
| Cm_for_mini_pUC(Cm)_rev | ATTATCTCATATTATAAAAGCCAGTCATTAG |
| TetR_pTripleTREP_in_for | ATTTCGTGAGTTGTTTAAACATTGATTAAGTACAAACTTGTG |
| TetR_pTripleTREP_in_rev | AATGGGATTTAGCTTCCTGACTCGCAC |
| P_TRE_ promoter and Twin-Strep-tag |  |
| pTRE_pTripleTREP_in_for | TCCCTTAACGTGAGTTGTACAATTCCCTATCAGTGATAGAGAAAAATTGTGAATTGACTCCCTATCAGTGATAGAGATATAATTAAGATAGATTCCCTATCAGTGAT |
| pTRE_pTripleTREP_in_rev | ATCACTGATAGGGAATCTATCTTAATTATATCTCTATCACTGATAGGGAGTCAATTCACAATTTTTCTCTATCACTGATAGGGAATTGTACAACTCACGTTAAGGGA |
| TwStr_pTripleTrep_in_for | TTCCCTATCAGTGATAGGGATCCTGGTCACATCCACAATTTGAAAAAGGTGGTGGTTCTGGTGGTGGTTCTGGTGGTTCAGCATGGTCACATCCGCAATTTGAAAAATAATGAGATCCTTCGTTCCACTGAGCGT |
| TwStr_pTripleTrep_in_rev | ACGCTCAGTGGAACGAAGGATCTCATTATTTTTCAAATTGCGGATGTGACCATGCTGAACCACCAGAACCACCACCAGAACCACCACCTTTTTCAAATTGTGGATGTGACCAGGATCCCTATCACTGATAGGGAA |
| pTripleTREP_linear_TwStr_for | TTCGTTCCACTGAGCGTCAG |
| pTripleTREP_linear_pTER_rev | AACTCACGTTAAGGGATTTTG |
| Terminators |  |
| ysnF_repC_in_for | ATTTAAATTTTGGAAATAGTGTAAAAACCTCAAATCCTAAATGGATTTGAGGTTTTTACATATTAATATTACTGAA |
| ysnF_repC_in_rev | TTCAGTAATATTAATATGTAAAAACCTCAAATCCATTTAGGATTTGAGGTTTTTACACTATTTCCAAAATTTAAAT |
| pTri_term_repC_for | ACATATTAATATTACTGAACAAAAATG |
| pTri_term_repC_rev | ACACTATTTCCAAAATTTAAATTCATG |
| yfhD_Cm_in_for | ATGACTGGCTTTTATAAGCAGCCGACAACAGAGCG |
| yfhD_Cm_in_rev | TGACAGATTATCTCATATTATCCTGACGAGGCGCC |
| Cm_Term_in_for | TATGAGATAATCTGTCAGACCAAG |
| Cm_Term_in_rev_new | TTATAAAAGCCAGTCATTAGGCCTATCTGAC |

**Table S2. Primers for the generation of vectors pTripleTREP_splA, pTripleTREP_splB and pTripleTREP_clpX.** Primers were synthesized by Biolegio (Netherlands).

| Primer | Sequence (5’-3’) |
| --- | --- |
| pTripleTREP_splA |  |
| splA_4_Trap_C-strep_for | TTCCCTATCAGTGATAGGGATACATTAACTATAAAAATAAATTTGGAAGGAGG |
| splA_4_Trap_C-strep_rev | TTGTGGATGTGACCAGGATCCTTTTTCAATATTATTTTGAATAAATTCTTTTAATTGTGGTGTG |
| splA_S189A_for | AGGTAATGCAGGATCTCCTGTATTG |
| splA_S189A_rev | AGATCCTGCATTACCTGGTTGTGC |
| pTripleTREP_splB |  |
| splB_4_Trap_C-strep_for | TTCCCTATCAGTGATAGGGACATCAATTCATTCGTGAAGTTG |
| splB_4_Trap_C-strep_rev | TTGTGGATGTGACCAGGATCCTTTATCTATGTTTTCTGCAATGAATTTTTTAATTTCTGGTG |
| splB_S193A_for | AAACGCTGGATCACCTGTATTAAAC |
| splB_S193A_rev | AGGTGATCCAGCGTTTCCGCTTTC |
| pTripleTREP_clpX |  |
| pTripleTREP_linear_TwStr_for | TTCGTTCCACTGAGCGTCAG |
| pTripleTrep_clpX_rev_long | CTTTTTACACCCCTATTTCCCTATCACTGATAGGGAATCTATC |
| clpX_TS_SD_for | AATAGGGGTGTAAAAAGAATGTTT |
| clpX_Term_pTripleTrep_rev | GCTCAGTGGAACGAAAAAAAAGCTCCGATCAAAGTTAAAC |

**Table S3. Primers for the generation of Northern Blot probes.** Primers were synthesized by Biolegio (Netherlands).

| Primer | Sequence (5’-3’) |
| --- | --- |
| *cat* probe (template: pIMAY) | |
| Cm_pIMAY_NOR_for | GGTGACAAGGGTGATAAACTC |
| Cm_pIMAY_T7_rev | GAATTAATACGACTCACTATAGGGAGACAGCAAGTTGAACTGAACCG |
| *splB* probe (template: *S. aureus* NCTC8325 gDNA) | |
| splB_for | ATTGGAACAACATTGGTTGAGGAAG |
| splB_rev_T7 | GAAATTAATACGACTCACTATAGGGAGAACTTTAATTCGCTCACCAGCTTTAG |

**Table S4. Information on reversed phase liquid chromatography (RPLC).**

| Instrument | UltiMate™ 3000 RSLCnano (Thermo Fisher Scientific, MA USA) |
| --- | --- |
| **Trap column** | 75 μm inner diameter, packed with 3 μm C18 particles (Acclaim PepMap100, Thermo Fisher Scientific) |
| **Analytical column** | Accucore 150-C18, (Thermo Fisher Scientific)  25 cm x 75 μm, 2,6 μm C18 particles, 150 Å pore size |
| **Buffer system** | binary buffer system consisting of 0.1% acetic acid in HPLC-grade water (buffer A) and 100% ACN in 0.1% acetic acid (buffer B) |
| **Flow rate** | 300 nl/min |
| **Gradient** | linear gradient of buffer B from 5% up to 25% 20min  0 min: 2%B  2min 5% B  10min 5% B  30 min: 25%B  35 min 40 %B  37 min 90 %B  49 min 90 %B  49,5 min 2 %B  50 min 2 %B |
| **Column oven temperature** | 40°C |

**Table S5 – Information on data dependent analysis (DDA) mass spectrometry.**

| Instrument | Orbitrap Exploris™ 480 (Thermo Fisher Scientific) |
| --- | --- |
| Operation mode | data-dependent |
| **Full MS** | |
| MS scan resolution | 120,000 |
| RF Lens (%) | 40 |
| Normalized AGC target | 300% |
| Microscans | 1 |
| Maximum ion injection time for the MS scan | Auto |
| Scan range | 350 to 1200 m/z |
| Polarity | positive |
| Spectra data type | profile |
| **Filter** | |
| MIPS | MIPS Mode=proteins, relaxed restriction when too few Precursors are found=True |
| Dynamic exclusion | exclude after n times = 1  Exclusion duration (s) = 10, exclude isotopes = True  Mass tolerance low = 10, high = 10 |
| Minimum Intensity | 5000 |
| Charge State | 2-6 |
| **Data dependent properties** | |
| Number of dependent scans | 20 |
| **dd-MS2** | |
| Resolution | 30,000 |
| MS/MS AGC target | 1e5 |
| Maximum ion injection time for the MS/MS scans | 22 ms |
| First mass (m/z) | 22 |
| Spectra data type | centroid |
| Isolation window | 1,4 m/z |
| Fixed first mass | 100 m/z |
| Normalized HCD collision energy | 30 % |
| RF Lens (%) | 50 |

**Table S6 – Plasmids with the same colE1 ori mutation as in pTripleTREP.** The nucleotide sequence of the colE1 ori finally present in pTripleTREP was searched using NCBI Nucleotide BLAST® and all accessions containing identical sequences were checked.

| Vector | NCBI Accession No | Reference |
| --- | --- | --- |
| pSD02 | MW263053 | (1,2) |
| pUC18 with inserted gDNA from N. benthamiana | HF675000 | (3) |
| pGMBsub03 | LT622642 | (4) |
| pJH1219 | OR348420 | Direct submission (J. Huang, 2023) |

Supplementary References

1. Dolinsky S, Haneburger I, Cichy A, Hannemann M, Itzen A, Hilbi H. The *Legionella longbeachae* Icm/Dot Substrate SidC Selectively Binds Phosphatidylinositol 4-Phosphate with Nanomolar Affinity and Promotes Pathogen Vacuole-Endoplasmic Reticulum Interactions. Infect Immun. 2014 Sep 10;82(10):4021–33.

2. Lam CN, Mehta-Kolte MG, Martins-Sorenson N, Eckert B, Lin PH, Chu K, et al. A Tail Fiber Engineering Platform for Improved Bacterial Transduction-Based Diagnostic Reagents. ACS Synth Biol. 2021 Jun 18;10(6):1292–9.

3. Sohn SH, Frost J, Kim YH, Choi SK, Lee Y, Seo MS, et al. Cell-autonomous-like silencing of GFP-partitioned transgenic *Nicotiana benthamiana*. J Exp Bot. 2014 Aug 1;65(15):4271–83.

4. Paracchini V, Petrillo M, Reiting R, Angers-Loustau A, Wahler D, Stolz A, et al. Molecular characterization of an unauthorized genetically modified *Bacillus subtilis* production strain identified in a vitamin B2 feed additive. Food Chem. 2017 Sep 1;230:681–9.
